# Melatonin: a ferroptosis inhibitor with potential therapeutic efficacy for the post-COVID-19 trajectory of accelerated brain aging and neurodegeneration

**DOI:** 10.1186/s13024-024-00728-6

**Published:** 2024-04-19

**Authors:** Asmaa Yehia, Osama A. Abulseoud

**Affiliations:** 1 Department of Neuroscience, Graduate School of Biomedical Sciences, Mayo Clinic College of Medicine, Phoenix, AZ 58054 USA; 2 Department of Medical Physiology, Faculty of Medicine, Mansoura University, Mansoura, Egypt; 3 Department of Psychiatry and Psychology, Mayo Clinic Arizona, 5777 E Mayo Blvd, Phoenix, AZ 85054 USA

## Abstract

The unprecedented pandemic of COVID-19 swept millions of lives in a short period, yet its menace continues among its survivors in the form of post-COVID syndrome. An exponentially growing number of COVID-19 survivors suffer from cognitive impairment, with compelling evidence of a trajectory of accelerated aging and neurodegeneration. The novel and enigmatic nature of this yet-to-unfold pathology demands extensive research seeking answers for both the molecular underpinnings and potential therapeutic targets. Ferroptosis, an iron-dependent cell death, is a strongly proposed underlying mechanism in post-COVID-19 aging and neurodegeneration discourse. COVID-19 incites neuroinflammation, iron dysregulation, reactive oxygen species (ROS) accumulation, antioxidant system repression, renin-angiotensin system (RAS) disruption, and clock gene alteration. These events pave the way for ferroptosis, which shows its signature in COVID-19, premature aging, and neurodegenerative disorders. In the search for a treatment, melatonin shines as a promising ferroptosis inhibitor with its repeatedly reported safety and tolerability. According to various studies, melatonin has proven efficacy in attenuating the severity of certain COVID-19 manifestations, validating its reputation as an anti-viral compound. Melatonin has well-documented anti-aging properties and combating neurodegenerative-related pathologies. Melatonin can block the leading events of ferroptosis since it is an efficient anti-inflammatory, iron chelator, antioxidant, angiotensin II antagonist, and clock gene regulator. Therefore, we propose ferroptosis as the culprit behind the post-COVID-19 trajectory of aging and neurodegeneration and melatonin, a well-fitting ferroptosis inhibitor, as a potential treatment.

## Introduction

Since December 2019, the coronavirus disease 2019 (COVID-19) pandemic has claimed millions of lives and left its extended mark on a large number of its survivors as a post-COVID syndrome [1]. Post-COVID syndrome, long COVID, or post-acute sequelae (PASC) of severe acute respiratory syndrome coronavirus 2 (SARS-CoV-2) infection, all refer to a multi-system affection including neurological, cognitive, and psychiatric manifestations that persist for more than 12 weeks after recovery from SARS-CoV-2 infection, regardless of illness severity, and cannot be explained by an alternative diagnosis [2–5]. The most common clinical manifestations include fatigue, anxiety, depression, headache, and cognitive impairment [6]. In a review of twelve studies, the overall prevalence of post-COVID-19 manifestations ranged from 35% to 90.5% [7]. The incidence of post-COVID syndrome six months after recovery from acute infection among 236,379 patients was 33.6% (95% CI: 33.17–34.07) and 46.4% (44.78–48.09) in patients who have been admitted to an intensive care unit [8]. The incidence of a first diagnosis of dementia in the 14 to 90 days after COVID-19 diagnosis was 1·6% (95% CI: 1.2–2.1) in people older than 65 years [9]. Another study from the same group assessed the risks of 14 neurological and psychiatric diagnoses after SARS-CoV-2 infection in 1,487,712 COVID-19 patients and compared these risks with the matched comparator cohort of patients with other respiratory infections. The authors reported that while the risks of common psychiatric disorders such as anxiety and depression returned to baseline after 1–2 months, the risks of cognitive deficits and dementia continued to increase at the end of the 2-year follow-up period [10]. Indeed, cognitive impairment is the single most reported complaint by patients with post-COVID syndrome [11–13]. What is more alarming than presenting with cognitive impairment is the potential of setting off a bleak trajectory of underlying accelerated brain aging and neurodegenerative disorders, which necessitates being equipped with knowledge of both the molecular underpinnings and the potential solutions. Here, we postulate a mechanistic hypothesis for the post-COVID-19 trajectory of aging and neurodegeneration. We present neuroinflammation, iron dysregulation, and ferroptosis, iron-dependent cell death, as a shared poisonous recipe. We portray melatonin in a new light and show how it fits perfectly as a ferroptosis inhibitor in such a predicament. We first discuss an overview of the underlying pathophysiology of ferroptosis. Next, we present the current evidence on the post-COVID-19 trajectory of brain aging and neurodegeneration. Finally, we provide a mechanistic hypothesis for melatonin as a ferroptosis inhibitor in the post-COVID-19 trajectory of aging and neurodegeneration.

## Ferroptosis

**Fig. 1:**
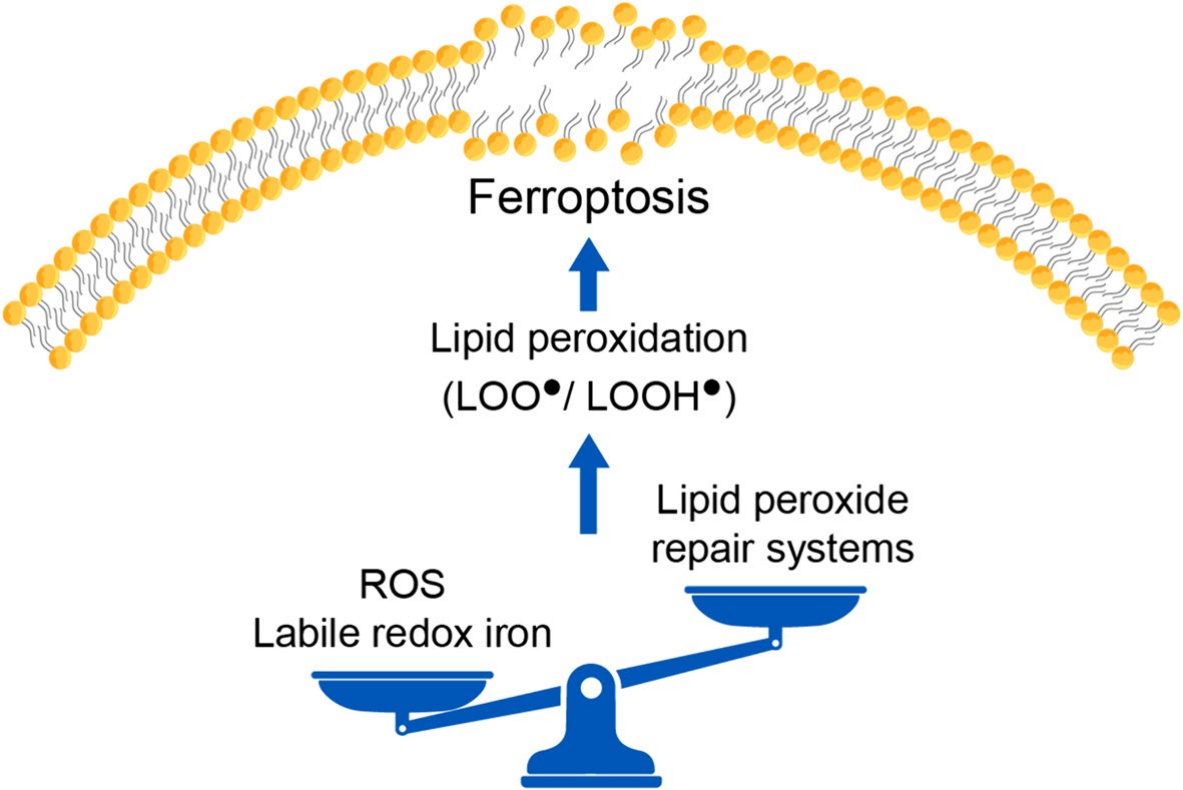
General theme of ferroptosis. Ferroptosis is an iron-dependent cell death that disrupts the integrity of cell membranes. Lipid peroxidation is the executer of ferroptosis with three main prerequisites: high levels of reactive oxygen species (ROS) and redox labile iron and compromised lipid peroxide repair systems. LOO^●^: lipid peroxides, LOOH^●^: lipid hydroperoxides

A decade ago, Dixon et al. coined the term ferroptosis after observing a genetically, morphologically, and biochemically distinctive cell death phenotype in cultured cancer cells and in glutamate-induced neuronal cell death. They introduced ferroptosis as an iron-dependent, non-apoptotic form of regulated cell death [14]. Ferroptosis, unlike other forms of cell death that need a direct trigger molecule, occurs as a result of an imbalance between the cellular oxidative media and antioxidant defense, which is fostered by labile reactive iron. Therefore, in all the variable pathways that end up in ferroptosis, three prerequisites are in order: iron accumulation in the redox (reduction–oxidation) state, accumulation of lipid peroxidation-induced reactive oxygen species (ROS), and failure of lipid peroxide repair systems [15] (Fig. 1). To view how these three factors condemn the cell to death, we will first discuss lipid peroxide repair and iron physiological machinery.

### Lipid peroxidation and Lipid peroxide repair machinery

**Fig. 2:**
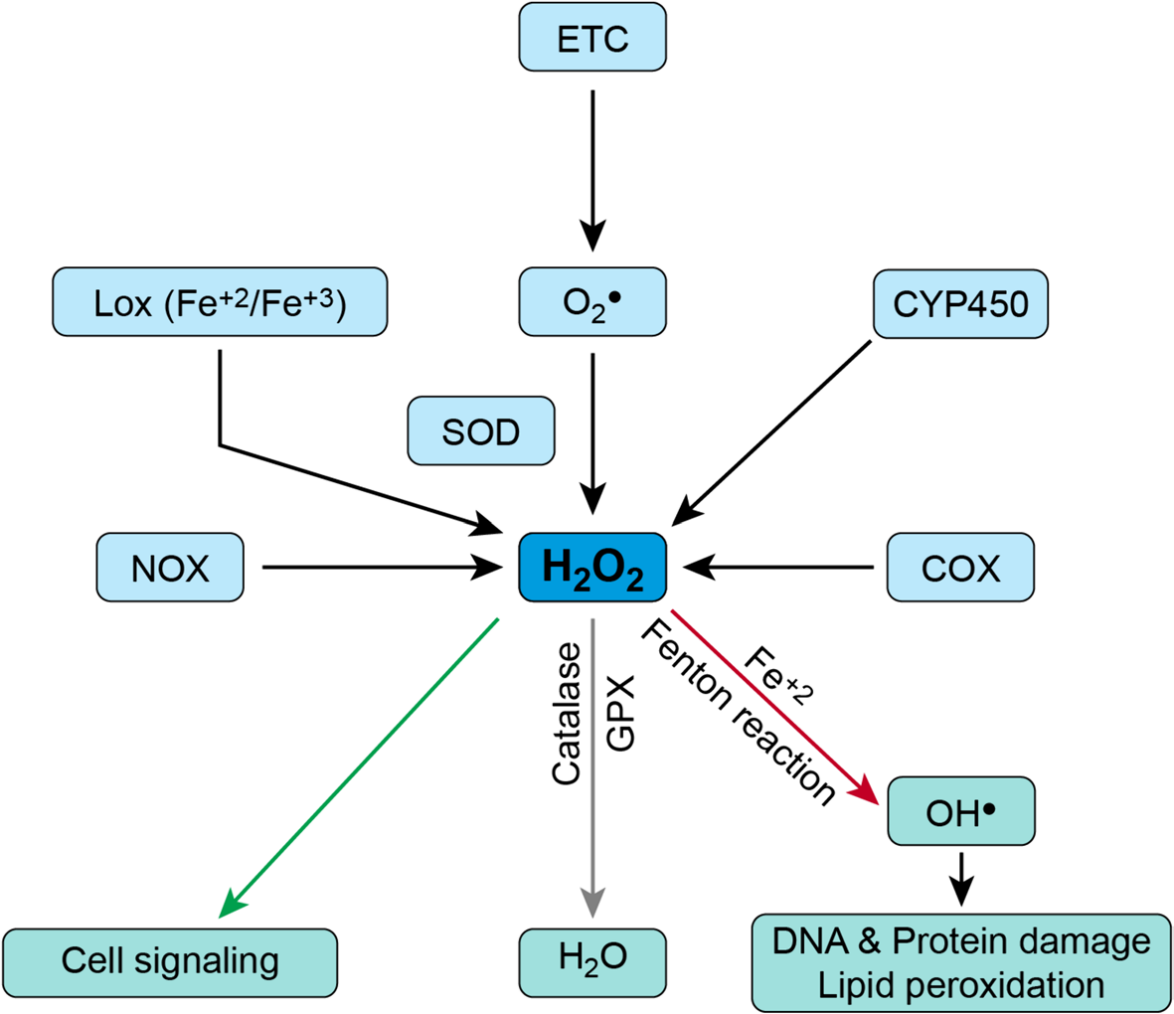
Sources and fates of Reactive oxygen species (ROS) as a main cause behind lipid peroxidation. Superoxide anion (O2^●^) is a chief radical produced normally during cell metabolism through the mitochondrial electron transport chain (ETC). Its oxidative effect is diffused by SOD (superoxide dismutase) which transforms it into the non-radical hydrogen peroxide (H2O2). H2O2 can also be enzymatically produced by nicotinamide adenine dinucleotide phosphate (NADPH) oxidases (NOXs), cytochrome P450 (CYP450), cyclooxygenases (COXs), and lipoxygenases (LOXs). H202 then can share in cell signalling processes or get neutralized through catalase and GPX (glutathione peroxidases). However, H2O2 can cause DNA and protein damage and lipid peroxidation through generating the highly reactive hydroxyl (OH^●^) radical. H2O2 generates OH^●^ radicals through the Fenton reaction by interaction with ferrous iron (Fe^+2^)

Ferroptosis renders cells a rather unique morphology, with mitochondrial size reduction, cell swelling, and plasma membrane thinning and rupture [16, 17]. This compromised cellular integrity at its core is executed by lipid peroxidation, which is fueled by redox-active iron and allowed to evolve in the absence of opposing lipid repair mechanisms [14, 15]. Reactive oxygen species (ROS) stand as the chief villain behind the process of lipid peroxidation [18]. ROS generically describes oxygen-derived oxidants that are actively engaged in redox reactions. They encompass both oxygen radicals and oxidizing non-radicals. A radical atom or molecule harbors one or more unpaired electrons (^●^), such as superoxide anion (O2^●^), hydroxyl (OH^●^), peroxyl (RO2^●^), and hydroperoxyl (HO2^●^) radicals. On the other hand, non-radicals such as hydrogen peroxide (H2O2) can easily generate highly reactive hydroxyl (OH^●^) radicals [19, 20]. H2O2 has poor reactivity to cellular biomolecules however, it can generate OH^●^ radicals through interaction with transition metals such as ferrous iron (Fe^+2^), which is called the Fenton reaction [21, 22]. ROS are indispensable to cellular homeostasis, mediating various processes of cell signaling and enzymatic regulation. They are normally produced during cellular metabolism, essentially through the mitochondrial electron transport chain and enzymatically through the transmembrane nicotinamide adenine dinucleotide phosphate (NADPH) oxidases (NOXs). Moreover, ROS can be generated by multiple other enzymes, including cytochrome P450, cyclooxygenases (COXs), and lipoxygenases (LOXs) [23]. However, once they surpass their physiological concentrations, ROS subject cells to oxidative stress and viciously interact with cellular proteins, carbohydrates, nucleic acid, and lipids, resulting in DNA and protein damage and lipid peroxidation [24] (Fig. 2).

**Fig. 3:**
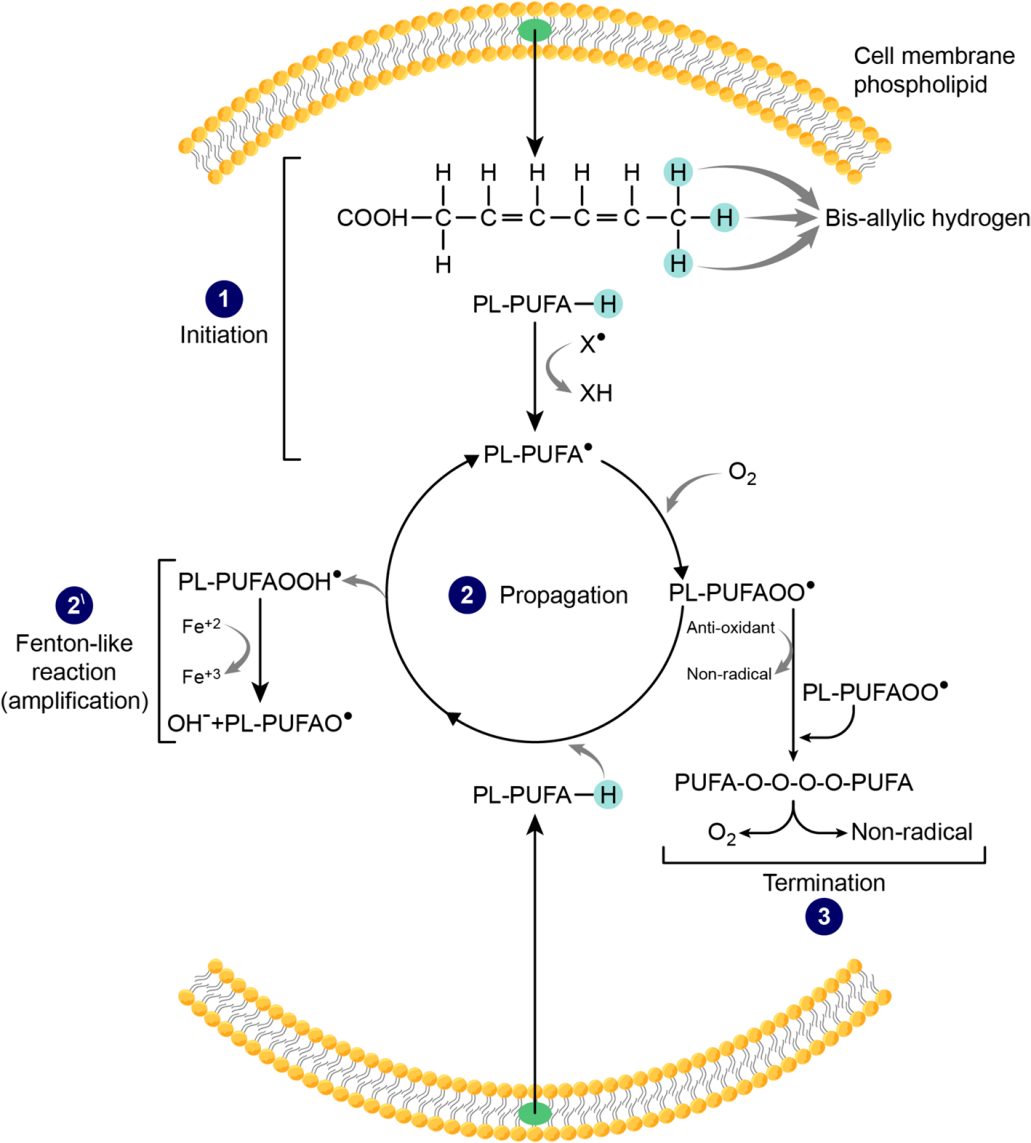
Mechanism of lipid peroxidation. Lipid peroxidation occurs in three steps: 1-initiation, 2-propagation, and 3-termination. 1- Initiation: the vicious cycle of lipid peroxidation starts with a radical attack (X^●^) which reacts with polyunsaturated fatty acids (PUFA) in cell membrane phospholipids (PL) and removes their bis-allylic hydrogen, transforming PUFA into a lipid/ alkyl radical (PL-PUFA^●^). 2- Propagation: Once a lipid radical is formed, a chain reaction of lipid radical formation continues. Molecular oxygen (O2) reacts with the formed PL-PUFA^●^, producing a lipid peroxyl radical (PL-PUFAOO^●^) which interacts with another membrane PL-PUFA, removes its bis-allylic hydrogen to form a lipid hydroperoxide radical (PL-PUFA-OOH^●^) and another PL-PUFA^●^ that propagates the cycle. 2^\^- Amplification: iron feeds this chain reaction through a Fenton-like reaction. Ferrous iron (Fe^+2^) reacts with PL-PUFA-OOH^●^, gets oxidized to Fe^+3^, and forms a hydroxide anion and a highly reactive alkoxyl (PL-PUFA-O^●^) radical that causes an exponential increase in lipid radical formation. 3- Termination: the cycle can be ended either by the presence of an antioxidant that turns radicals into non-radicals or by the interaction of two radicals, forming O2 and a non-radical

Lipid peroxidation refers to lipid oxidative degradation as a result of the interaction of oxygen with unsaturated fatty acids and the formation of lipid peroxides (LOO^●^) and lipid hydroperoxides (LOOH^●^), which triggers a chain reaction of lipid radical formation [25]. Cell membranes are most vulnerable to lipid peroxidation due to their abundance of polyunsaturated fatty acids (PUFA)-containing phospholipids (PL). PUFA have more than one double-bonded carbon in their hydrogen-carbon backbone and, therefore, several bis-allylic hydrogen atoms [26]. Bis-allylic hydrogen is the one attached to the carbon adjacent to two double-bonded/unsaturated carbon atoms. Bis-allylic hydrogen forms the weakest hydrogen-carbon bond, which makes its removal an easy task and renders PUFA more susceptible to radicals’ attacks [27, 28]. Lipid peroxidation, as a chain reaction of radicals’ formation, occurs in three steps: initiation, propagation, and termination. Lipid peroxidation is initiated by a radical attack such as OH^●^ or HO2^●^ radicals, which remove the bis-allylic hydrogen from the PUFA chain and generate a carbon-centered lipid radical or alkyl radical (PL-PUFA^●^). Once a lipid radical is formed, the reaction snowballs to a detrimental fate where PL-PUFA^●^ interacts with molecular oxygen, forming a lipid peroxyl radical (PL-PUFAOO^●^). PL-PUFAOO^●^ then abstracts a hydrogen from an adjacent PL, forming a lipid hydroperoxide radical (PL-PUFA-OOH^●^) and a new lipid radical, which helps propagate the chain reaction [29–32] (Fig. 3). Lipid peroxidation and PL-PUFA-OOH^●^ formation can occur without or with an enzymatic intervention by cytochrome P450 oxidoreductase, COXs, and LOXs enzymes [33]. LOXs can be considered an indirect manifestation of iron’s role in ferroptosis since their activity requires the oxidation of ferrous (Fe^+2^) to ferric (Fe^+3^) iron [34]. The involvement of either enzymatically or non-enzymatically formed PL-PUFA-OOH in ferroptosis is still a controversy [35, 36]. Iron can feed this malicious cycle, producing more lipid radicals through a Fenton-like reaction. The Fenton reaction is a series of redox reactions catalyzed by the labile Fe^+2^ pool. Fe^+2^ reacts with PL-PUFA-OOH^●^, gets oxidized to Fe^+3^, and forms a hydroxide anion and a highly reactive alkoxyl (PL-PUFA-O^●^) radical [37, 38]. During the propagation step, the sequential production of more lipid radicals incites exponentially growing damage. The chain reaction can continue, damaging the cell membrane lipid bilayer [39]. Along this process, highly toxic lipid peroxidation degradation products such as malonaldehyde (MDA) and 4-Hydroxynonenal (4-HNE) are formed and react with cell proteins and DNA, altering their structure and function and fostering a state of cytotoxicity [40–42]. Finally, the termination step can come about by quenching radicals by an anti-oxidant or reacting with another radical [31, 43] (Fig. 3).

**Fig. 4:**
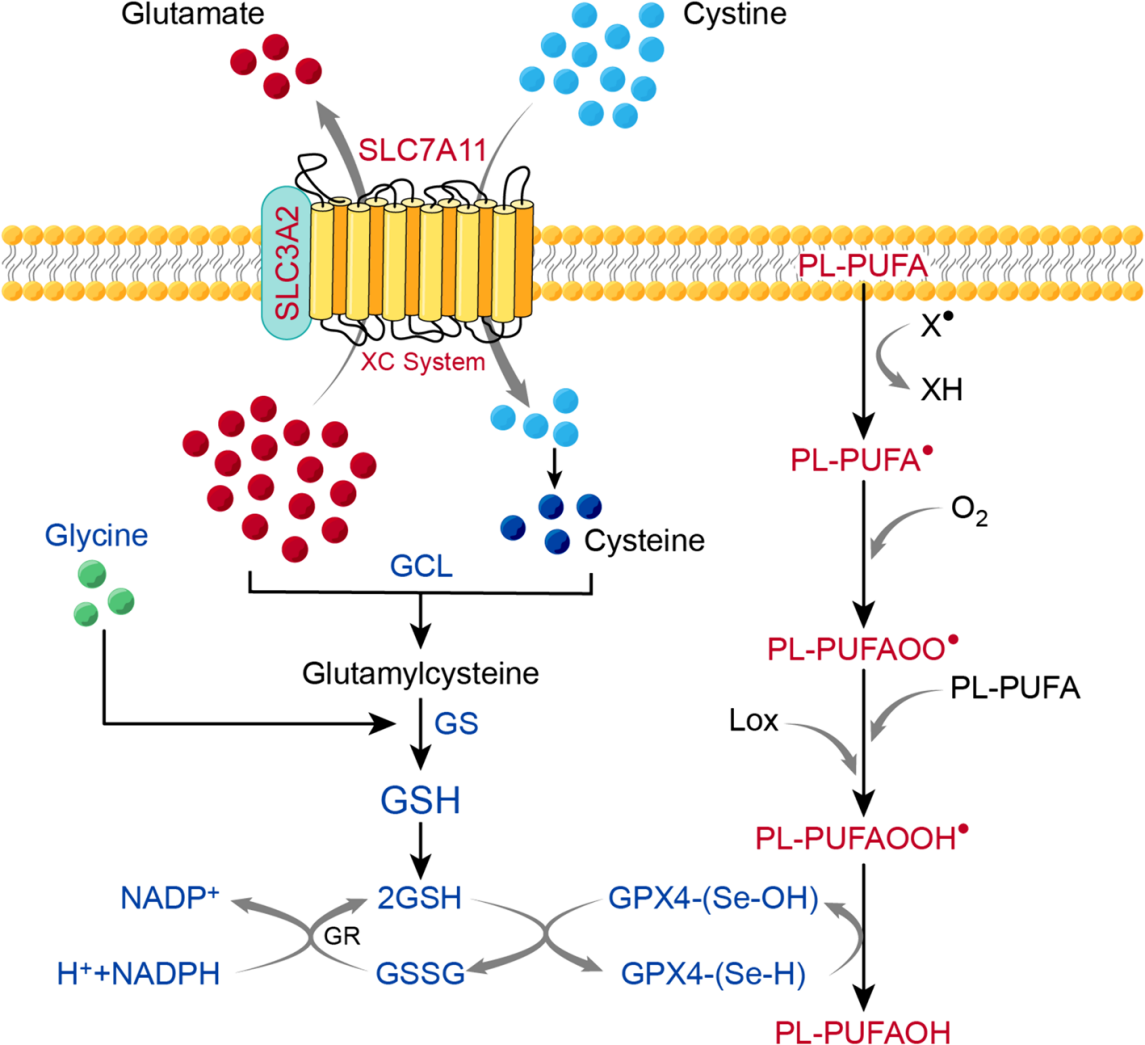
Glutathione (GSH)-glutathione peroxidase 4 (GPX4) lipid peroxide repair system. GSH is a tripeptide formed of; cysteine, glutamate, and glycine. Cysteine is formed from cystine that enters the cell through a cystine/glutamate antiporter or Xc − system. The Xc- system is formed of a heavy chain subunit called Solute carrier family 3 member 2 (SLC3A2) and a light chain subunit called xCT, or Solute carrier family 7 member 11 (SLC7A11). The antiporter allows cystine into the cell in exchange of glutamate exiting the cell, with both moving down their concentration gradient. Once entered the cell, cystine is transformed into cysteine which binds to glutamate by the glutamate-cysteine ligase (GCL) forming a glutamylcysteine dipeptide. Glycine is added to the dipeptide to form GSH by glutathione synthase (GS). GPX4 interrupts lipid peroxidation by reducing lipid hydroperoxides (PL-PUFAOOH^●^) to their corresponding alcohols (PL-PUFAOH). To do so, the active site of GPX4, selenol (Se-H), gets oxidized to selenenic acid (Se-OH). To restore the reducing capacity of GPX4, GSH reduces the selenenic acid back to the active selenol and gets oxidized to glutathione disulfide (GSSG). To restore the reducing capacity of GSH, GSSG (the oxidized form) is turned into 2 GSH (the reduced form) by glutathione reductase (GR), using Nicotinamide Adenine Dinucleotide Phosphate Hydrogen (NADPH) as a reducing agent. PL-PUFA: phospholipid polyunsaturated fatty acids, PL-PUFAOO^●^: lipid peroxyl radical

Under normal conditions, cells can counterbalance these events through their antioxidant defense systems. Antioxidant cell defense works on the scavenging and removal of active oxidants [33] with multiple antioxidant enzymes, including superoxide dismutase (SOD), catalase, and peroxidases [28]. One of the most dedicated phospholipid repair systems is the glutathione (GSH)-glutathione peroxidase 4 (GPX4) system. The GPX4 enzyme, or phospholipid hydroperoxide GPX (PHGPX), is an oxidoreductase that belongs to a family of eight GPXs [44]. GPX4 is a selenoprotein owing to its constituent selenocysteine, the 21st amino acid, which resembles cysteine (Cys) but differs by having a single atom of selenium instead of sulfur [45]. GPX4 can efficiently reduce hydroperoxides (OOH^●^) to their corresponding alcohols (-OH). GPX4 can execute the oxidation/reduction steps by shifting its selenocysteine active site between an oxidized and a reduced state. First, GPX4 reduces the toxic lipid peroxides to non-toxic lipid alcohols by oxidizing its active site, selenol (Se-H), to selenenic acid (Se-OH). At this point, the reducing GSH steps in to reduce the selenenic acid back to the active selenol, allowing the repeating of the oxidation/reduction process, and then it gets oxidized to glutathione disulfide (GS-SG) [46]. GSH is the most abundant cellular antioxidant. GSH is a tripeptidic thiol (sulfur containing), formed of cysteine, glutamate, and glycine. The glutamate and cysteine amide bond is catalyzed by glutamate cysteine ligase (GCL), then glycine is added to the dipeptide to form GSH by glutathione synthase. The sulfur-containing cysteine renders GSH strong reducing and electron-donating properties, and is considered a rate-limiting substrate in the synthesis of GSH [47–49]. GSH alternates from its reduced form (GSH) to its oxidized glutathione disulfide form (GSSG), which turns back to GSH by glutathione reductase. Once GSH loses electrons to become oxidized, GSSG is formed by a disulfide bond between two GSH molecules [50, 51]. A critical component for the GSH/GPX4 system to fulfill its role is the Cystine/Glutamate antiporter or system Xc-. System Xc- is crucial for GSH availability [52]. It is formed of a 4F2 heavy chain (4F2hc) or Solute carrier family 3 member 2 (SLC3A2) subunit and a light chain subunit called xCT, or Solute carrier family 7 member 11 (SLC7A11). System Xc- allows the transport of both cystine and glutamate in either direction. However, based on their concentration gradient and glutamate being shuttled intracellularly by its own transporter, the excitatory amino acid transporter (EAAT), glutamate is exported and cystine is imported. Cystine, the oxidized form of cysteine, can then be reduced to cysteine by GSH or thioredoxin reductase 1 (TRR1) [53, 54] (Fig. 4). Another judging factor for the availability and biosynthesis of GSH is the Nuclear factor erythroid 2-related factor 2 (NrF2) which is the major transcriptional factor that governs the expression of GSH and its related transporters among multiple anti-oxidant cellular responses [55, 56]. NrF2 promotes the expression of GCL, GR, and GPX4 and directly activates the SLC7A11 subunit of system Xc-. NrF2 is repressed by the Kelch-like ECH-associated protein 1 (Keap1), which is responsible for NrF2 regulation. In the case of oxidative stress, the redox-sensitive Keap1 is inactivated which allows the translocation of NrF2 into the nucleus to fulfill its role [57, 58].

### Iron physiological machinery

Iron is a peculiar metal that has a remarkable dichotomy of serving essential physiological processes for cell survival and, on the other hand, driving cells to their demise once tipped over its sensitive balance [59, 60]. Due to the toxic capacity of iron to overwhelm the antioxidant systems with ROS once free and overloaded, several proteins cater for iron, rendering most body iron protein bound. The portion of iron that is protein-unbound is referred to as labile iron, which possesses redox characteristics (the ability to undergo reduction-oxidation reactions). Also, it is the form of iron that is exchangeable and chelatable [61].

The lack of a physiological mechanism for iron excretion adds another reason, besides its toxic propensity, to subject iron to strict regulation. The systemic iron pool builds up mainly from duodenal enterocytes absorbing iron, hepatocytes, and macrophages of the reticuloendothelial system recycling senescent erythrocytes, with ferroportin (FPN1) as the only way out to exit these cells. FPN1 is strictly controlled by hepcidin, a 25-amino acid peptide produced by the liver. Hepcidin expression, under physiological conditions, is upregulated by high serum iron. Hepcidin is considered the master hormonal regulator of iron since it controls the systemic availability of iron by limiting its intestinal absorption and blocking its cellular release through FPN1 degradation [62–65]. In the intestinal lumen, duodenal cytochrome b (Dcytb), on the apical border of enterocytes, reduces Fe^+3^ to Fe^+2^, which is transported into the cytoplasm through the divalent metal transporter 1 (DMT1) [66]. Fe^+2^ can then exit the basolateral border of enterocytes into the circulation through FPN1. Exported Fe^+2^ is oxidized by hephaestin or ceruloplasmin to form Fe^+3^ [67]. Fe^+3^ is transported within the circulation bound to liver-synthesized transferrin that allows iron entry into cells through transferrin receptors (TfR1). The TfR1-transferrin complex is then taken up into the cell by clathrin-mediated endocytosis. In the endosome, the ferrireductase activity of the six-transmembrane epithelial antigen of prostate 3 (STEAP3) reduces Fe^+3^ to Fe^+2^ [68]. Fe^+2^ is released from the endosome into the cytoplasm through DMT1 and the transient receptor potential mucolipin 1 (TRPML1). In addition, DMT1 on cell membranes can allow the passage of non-transferrin-bound iron (NTBI) [69]. Iron can then be utilized, exported, or stored in the form of ferritin. Apoferritin is an iron storage shell formed of 24 subunits of two types: Heavy (H ferritin) and Light (L ferritin). Iron loading into apoferritin is mediated by H ferritin, which has ferroxidase enzymatic activity to oxidize Fe^+2^ to Fe^+3^ [63, 70]. On the other hand, iron mobilization from ferritin is facilitated by ferritinophagy. Ferritinophagy is an autophagic degradation of ferritin mediated by nuclear receptor co-activator 4 (NCOA4), increasing iron cellular availability [71, 72]. In cases of excess labile catalytic iron pool and over-activated ferritinophagy, H ferritin steps in to load iron into ferritin to prevent triggering ferroptosis [73, 74]. Serum ferritin, secreted from hepatocytes, reticuloendothelial cells, and other parenchymal cells, has always been an indicator for body iron stores since it correlates with intracellular iron concentration in physiological settings and in cases of iron overload [75–77].

**Fig. 5:**
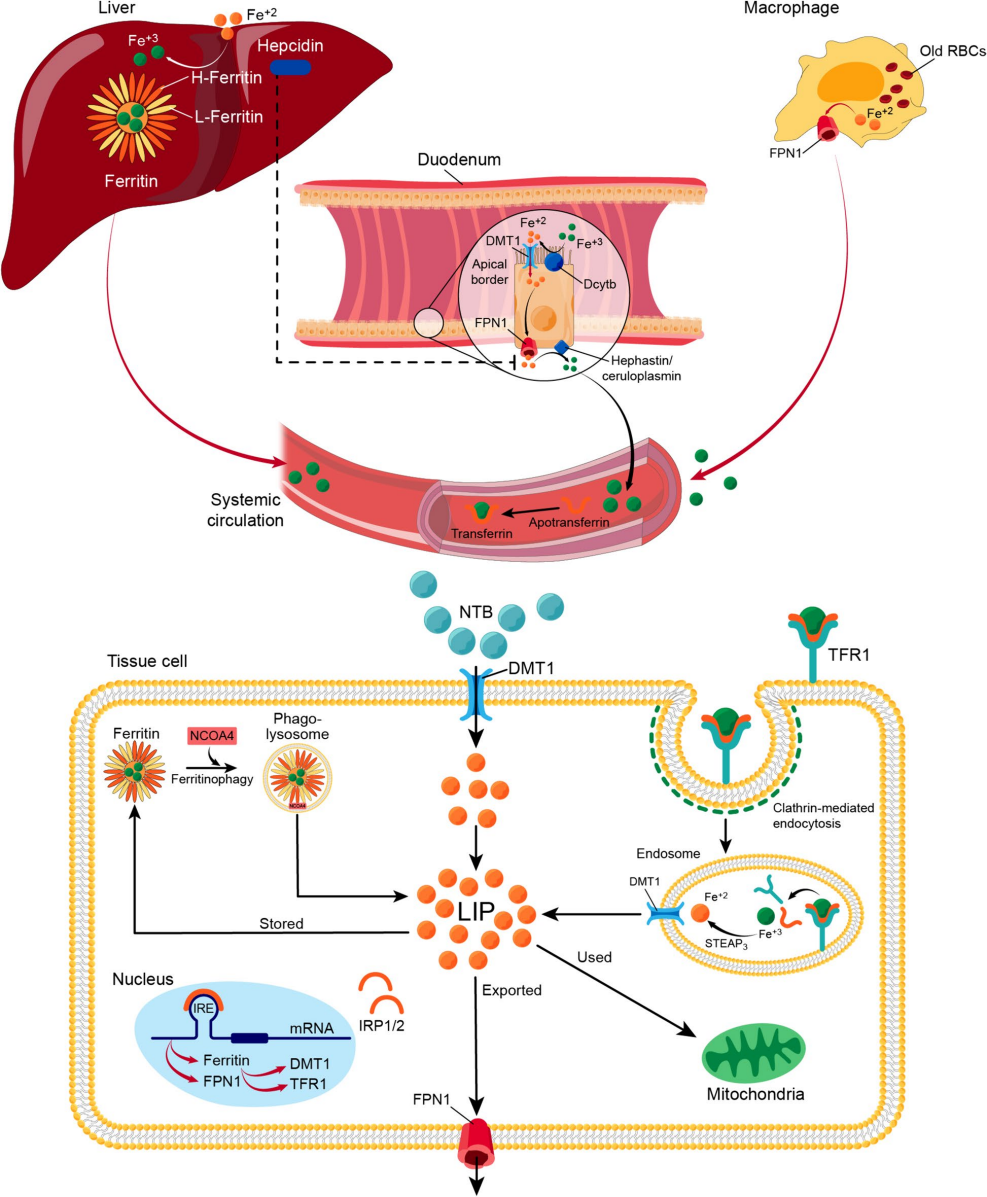
Iron physiological machinery. The availability of iron in the systemic circulation depends on its absorption from the duodenum, release of iron from old red blood cells (RBCs) engulfed by macrophages, and release of iron from its liver stores, stored as ferritin. Ferritin is an iron storage shell formed of 2 type subunits: Heavy (H ferritin) and Light (L ferritin). H ferritin has a ferroxidase enzymatic activity to oxidize ferrous (Fe^+2^) to ferric iron (Fe^+3^) and load it into the shell. In the duodenum, duodenal cytochrome b (Dcytb), on the apical border of enterocytes, reduces Fe^+3^ to Fe^+2^ to enter the cell through the divalent metal transporter 1 (DMT1). Fe^+2^ then exit enterocytes through ferroportin 1 (FPN1). FPN1 is under the control of hepcidin, the only hormonal regulation of iron. Exported Fe^+2^ is oxidized by hephaestin or ceruloplasmin to form Fe^+3^. Within the circulation, Fe^+3^ binds to apotransferrin, forming transferrin. Iron then gets distributed to body systems and each cell forms its share of labile iron pool (LIP). The LIP can be formed from three routes. First, the release of iron from its stores in ferritin by the process of ferritinophagy mediated by nuclear receptor co-activator 4 (NCOA4). Second, imported into the cell as non-transferrin-bound (NTB) iron through DMT1. Third, imported into the cell as transferrin which binds to transferrin receptors-1 (TfR1). The TfR1-transferrin complex enters the cell by clathrin-mediated endocytosis. In the endosome, the six-transmembrane epithelial antigen of prostate 3 (STEAP3) reduces Fe^+3^ to Fe^+2^ by its ferroxidase activity. Then, Fe^+2^ is released into the cytoplasm through DMT1. Once in the cell, iron can then be utilized, exported through FPN1, or stored in the form of ferritin. Iron-related proteins are under a tight regulation of a molecular machinery in the form of the iron responsive element/iron regulatory protein (IRE/IRP) system. IRP1 and IRP2 bind to IRE in the mRNA of different target genes and control their translation, including TfR1, DMT1, ferritin, and FPN1

With iron being utilized or stored in the form of ferritin, the free iron existing in the labile iron pool, the redox active iron, remains a concern that needs regulation, especially with the previously mentioned role of iron in promoting lipid peroxidation [61, 78]. To guarantee tight regulation, molecular machinery for the regulation of iron transporters exists in the form of the iron responsive element/iron regulatory protein (IRE/IRP) system. IRP1 and IRP2 are cytosolic proteins that bind to IRE in the mRNA of different target genes and control their translation, including TfR1, DMT1, ferritin, and FPN1 [79]. Once the IRPs are activated, they promote the expression of TfR1 and DMT1 and inhibit that of FPN1 and ferritin, which favors the increment of the labile iron pool and a potential unfavorable outcome [80, 81] (Fig. 5).

### Overview of ferroptosis molecular machinery

**Fig. 6:**
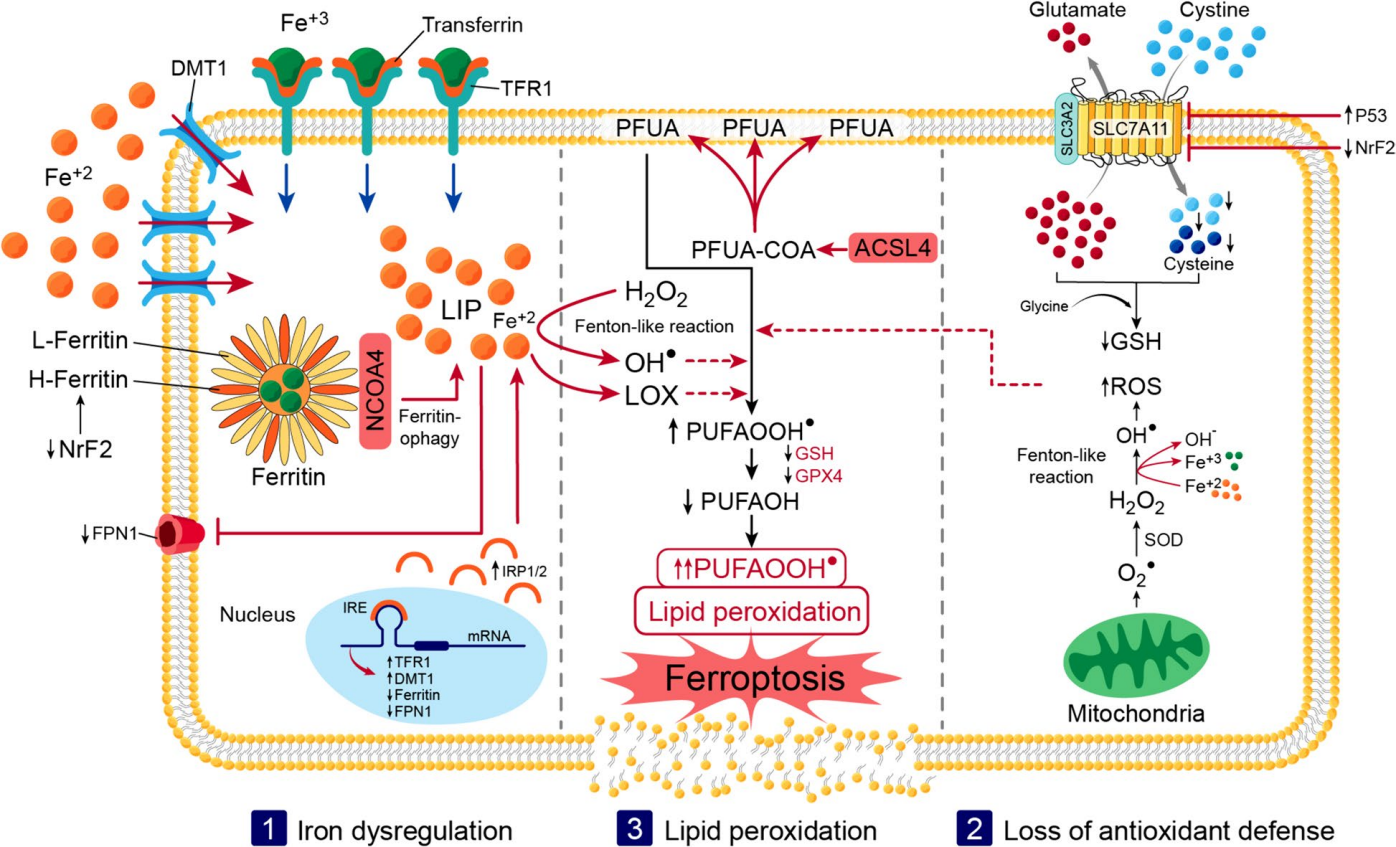
Ferroptosis molecular machinery. Ferroptosis is an iron-dependent cell death which is executed by lipid peroxidation. The loss of iron regulation and functioning lipid peroxide repair systems foster lipid radical production and lipid peroxidation. 1- Iron dysregulation: During ferroptosis, there is an increase in the labile iron pool (LIP), which is the redox active iron. Within cells, the LIP increases through importing more iron through the upregulated divalent metal transporter 1 (DMT1) and transferrin receptors-1 (TFR1), limiting iron exportation by degrading FPN1, and releasing iron from its ferritin stores by the nuclear receptor co-activator 4 (NCOA4)- mediated ferritinophagy or ferritin degradation. At a molecular level, iron regulatory proteins (IRP1/2) are upregulated and bind to the iron responsive element (IRE) in the mRNA of iron-related proteins, increasing the the expression of TfR1 and DMT1 and inhibiting that of FPN1 and ferritin, which favors the build-up of LIP. 2-Loss of antioxidant defense: Through a Fenton-like reaction, ferrous iron (Fe^+2^), in the expanding LIP, acts on hydrogen peroxides (H2O2) to form highly reactive hydroxyl (OH^●^) radicals. The accumulation of reactive oxygen species (ROS) overwhelms the already-inflicted antioxidant defence system. During ferroptosis, nuclear factor erythroid 2-related factor 2 (NrF2) decrease and P53 increase can downregulate of the Solute carrier family 7 member 11 (SLC7A11), which denies the entry of cystine and therefore decrease the formation of glutathione (GSH). 3- Lipid peroxidation: During ferroptosis, an increase in acyl-coenzyme A synthetase long-chain family member 4 (ACSL4) incorporates more polyunsaturated fatty acids (PUFA) into membrane phospholipid by binding them to coenzyme A to produce PUFA-CoAs. The abundance of ROS initiates a chain reaction of lipid peroxidation forming lipid hydroperoxides (PUFAOOH^●^). The chain reaction is amplified by the grown LIP and lipoxygenases (LOX) that help the enzymatic formation of PUFAOOH^●^. With the decreased availability of glutathione peroxidase 4 (GPX4) and GSH. The chain reaction of lipid peroxidation propagates extensively, inciting more and more ferroptosis

Ferroptosis can be described as a state of “loss of control” of both lipid repair systems and iron in the presence of inflated lipid peroxidation. Lipids comprise around 50% of the brain's dry weight [82], which makes the brain an easy target [83]. Since lipid peroxidation is considered the core event in ferroptosis, the higher the content of PUFA, the more sensitive cells are to ferroptosis [84]. In a study in germ cells, the dietary ingestion of the PUFA dihomo-γ-linolenic acid induced ferroptosis [85]. Moreover, the administration of arachidonic acid, an omega-6 PUFA, combined with interferon gamma (IFN‐γ), mediated ferroptosis in tumor cells in an acyl-coenzyme A synthetase long-chain family member 4 (ACSL4)-dependent manner [86]. ACSL4 is a crucial enzyme for incorporating PUFA into PL by binding them to coenzyme A to produce PUFA-CoAs [87]. Therefore, ACSL4 can induce ferroptosis by changing the landscape of existing lipids with a more robust content of PUFA-containing PL [88]. What can also induce ferroptosis through changing the lipid landscape, in terms of higher lipid peroxide formation, are NOXs and LOXs enzymes [36] (Fig. 6).

Failure of lipid peroxidation defenses takes up a noticeable role in ferroptosis induction. The inactivation or deletion of GPX4, as an essential lipid peroxidation opponent, is extensively reported and reviewed as a competent tactic for ferroptosis induction in various cell types [89–91]. In addition, GSH depletion is another way to halt the GPX4/GSH protective effect against ferroptosis. GSH depletion can be a consequence of the inactivation of SLC7A11 of system Xc- which impairs the importing of cystine and hence the available cysteine needed for GSH synthesis [15]. The expression of SLC7A11 is regulated by multiple transcriptional factors, such as tumor protein p53 (p53) [92] and NrF2 [58]. P53 is a tumor suppressor that is activated during cellular stress and can induce cell cycle arrest and senescence. P53 suppresses SLC7A11 expression and enables the induction of ferroptosis [92]. On the other hand, the anti-oxidant transcriptional factor NrF2 can redeem the expression of SLC7A11 and therefore form an opposing force against ferroptosis [93].

During ferroptosis, the tight regulation of iron falls into complete chaos, which amplifies the labile iron pool and its threatening redox activity. The upregulation of TfR1 feeds the labile iron pool with more iron uptake; therefore, TfR1 high expression is a prominent feature of ferroptosis [94, 95]. Another way to feed the labile iron pool is through the overexpression of DMT1, which also works as a ferroptosis mediator [96, 97]. The same fate is fulfilled with the upregulation of IRPs [98, 99], the downregulation of FPN1 [100, 101], and the increased production of hepcidin, trapping more iron within cells [102]. The autophagic degradation of ferritin, NCOA4-mediated ferritinophagy, disrupts the ratio of ferritin-stored iron to free redox-active iron in favor of the latter [103]. The reduction of NrF2 was found to aggravate NCOA4 ferritinophagy [104]. Moreover, NrF2 helps up-regulate H ferritin expression and, hence, iron loading into ferritin, shielding cells from ferroptosis [105] (Fig. 6). Besides ferritinophagy, another autophagic degradation of the clock gene transcriptional factors, termed clockophagy, can also mediate ferroptosis [106].

## Post-COVID-19 trajectory of brain aging and neurodegeneration

The inquiry of whether COVID-19 impacts the brain to the point of accelerating the physiological aging process stems from various intriguing findings. Aging was repeatedly reported as an imperative risk factor for a deteriorated course of COVID-19 infection [107, 108]. Senescent cells, positive for the cell cycle inhibitor p16, were more prevalent in the postmortem frontal cortex of patients with severe SARS-CoV-2 infection compared to their age-matched controls. Senolytic drugs such as dasatinib and quercetin have shown an improved SARS-CoV-2 clinical course and survival in angiotensin converting enzyme 2 (ACE2) receptors- overexpressing mice and in patients [109, 110]. Tripathi et al. also found senescence in human non-senescent cells and exaggeration of the senescence-associated secretory phenotype (SASP)-related inflammatory molecules in human senescent cells with the application of the SARS-CoV-2 spike protein [111]. Similar findings were reported by Meyer et al. where they found enhanced expression of senescence-related markers, p16, p21, and β-galactosidase (SA-β-Gal), in spike-transfected human epithelial cells with increased release of SASP, which was reversed by inhibiting interleukin (IL)-6 signaling [112]. The argument about COVID-19-associated aging could be further validated by the depicted telomere shortening and DNA methylation in a cohort of 407 COVID-19 patients during the initial phase of the disease [113].

The emergence and acceleration of neurodegenerative disorders post-COVID-19 infection is a recurring observation. Patients with Parkinson’s disease (PD) reported the occurrence or exacerbation of both motor and non-motor symptoms such as mood changes, sleep disorders, and cognitive impairment [114, 115]. Another alarming observation is the higher mortality rates of PD patients post-COVID-19 compared to the pre-COVID-19 rates, with PD shown as a risk factor for COVID-19-associated mortality [116, 117]. Also, patients with PD displayed a higher susceptibility to SARS-CoV-2 infection when compared to their matched controls [118]. A similar scenario unfolds in the case of Alzheimer's disease (AD), where a higher risk of new-onset AD in COVID-19-infected patients is reported compared to their matched controls [119]. AD patients are also more susceptible to SARS-CoV-2 infection, with AD being a risk factor for a poor prognosis of COVID-19 infection [120]. Deterioration of neuropsychiatric manifestations and cognitive decline in AD patients has been a frequent observation during the COVID-19 pandemic [121]. Frontera et al. interestingly, reported higher neurodegenerative markers (neurofilament light chain (NfL), ubiquitin carboxy-terminal hydrolase L1 (UCHL1), and glial fibrillary acidic protein (GFAP)) in COVID-19 patients when compared to non-COVID controls with AD or with mild cognitive impairment [122], implying a deteriorated prognosis for neurodegenerative disorders with COVID-19 infection. The SARS-CoV-2 spike protein receptor-binding domain (RBD) binds to several proteins, such as amyloid β (Aβ) and tau, accelerating their brain aggregation [123]. One year post-COVID-19, Ferrucci et al. detected brain hypometabolism and Aβ deposition in a positron emission tomography (PET) scan of one patient out of 7 patients with cognitive impairment [124], which mandates further investigation.

Aligning with the plausible development of accelerated aging or neurodegenerative disorder, cognitive impairment is highly prevalent among patients with post-COVID syndrome [8, 10]. Several studies assessed alterations in specific cognitive domains (i.e., executive functions, speed of processing, memory, attention, and language) [reviewed in [11–13] with attention deficits as the most common affected domain [125–130]. Liu et al. reported significant cognitive impairment in hospitalized COVID-19 older adults (*n* = 1,539) compared to a healthy control group (*n* = 466) six months after hospital discharge [131]. Matias-Guiu et al. utilized unsupervised machine-learning clustering algorithms to cluster cognitive domains impaired in a cohort of patients with post-COVID syndrome (*n* = 404) in comparison to healthy controls (*N* = 145) and reported that as many as 41.2% of the sample were classified as having at least one cognitive domain impaired, mostly attention and processing speed [125]. Another study reported that 85% of patients with post-COVID-19 syndrome (*n* = 214) had some alterations in at least one cognitive domain, with attention and executive functions being the most frequently affected areas [126]. Similar results were reported by Garcia-Sanchez et al., who also identified deficits in the attention domain as the most prominent cognitive impairment (with and without associated executive deficits) in a sample of post-COVID-19 patients with subjective cognitive complaints (*n* = 63) [127]. Delgado-Alonso et al. reported attention deficits associated with poor performance in executive function, working, and episodic memory, as well as visuospatial processing, in a group of post-COVID-19 patients (*n* = 50) with subjective cognitive complaints [132]. Unfortunately, cognitive impairment has been documented in asymptomatic or mildly symptomatic SARS-CoV-2 individuals [130], non-hospitalized COVID-19 patients [128], and severe, critically ill patients [133], with evidence that COVID-19 patients with severe illness have worse cognition compared to non-severe patients and to healthy controls [131, 133]. The menacing yet enigmatic nature of these observations demands robust research efforts dedicated to investigating the underlying mechanisms and providing satisfactory solutions. Therefore, we present ferroptosis as a mechanism behind the post-COVID trajectory of aging and neurodegeneration and melatonin as a well-fitted answer. We first discuss the physiological background of melatonin and how it could be the answer as an anti-ferroptosis.

## Melatonin

### Physiology of melatonin

More than half a century ago, melatonin was identified as the chief hormone of the pineal gland. The name came from its observed capacity to lighten fish and frogs, skin color by shielding melanocytes from the darkening effect of the melanocyt-stimulating hormone and gathering melanin granules [134, 135]. Therefore, the name came about as melanophore-contracting hormone from the Greek; melas meaning "dark" and “tonos” meaning to suppress [136]. Ironically, later, meltonin became popular as the “darkness hormone” [137] when its versatile biological effects started to unfold and it was recognized as the main regulator of the body’s circadian dark/light cycle [138–141].

#### Synthesis and release

**Fig. 7:**
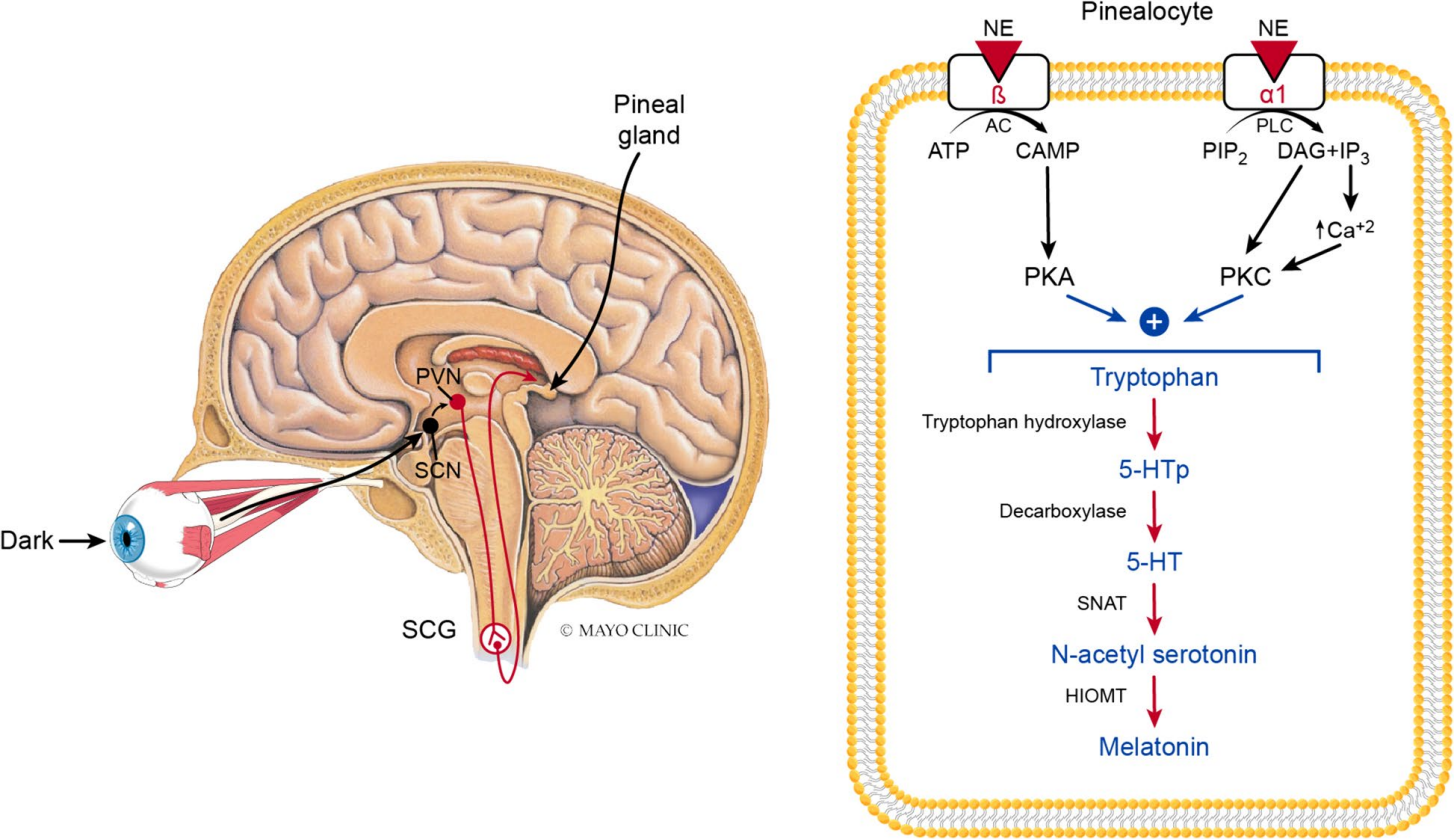
Melatonin’s synthesis and release. Melatonin is a dark hormone where its synthesis and release from the pineal gland depend on the the the dark/light signal it receives from the “central biological clock”, the suprachiasmatic nucleus (SCN) in the hypothalamus. The SCN receives light information through the retinal-hypothalamic tract from retinal ganglionic cells and then sends adrenergic sympathetic fibers to the pineal gland after synapsing in the paraventricular nucleus (PVN) of the hypothalamus and the superior cervical ganglia (SCG). The post-ganglionic sympathetic fibers releases noreadrenaline (NE), which activates the pinealocyte ß and α1 adrenergic receptors. The activation of ß receptors activates adenyl cyclase (AC), which increases cyclic adenosine monophosphate (cAMP) and protein kinase A (PKA). The activation of α1-adrenergic receptors activates phospholipase C (PLC) which acts on Phosphatidylinositol 4,5-bisphosphate (PIP2) to form 1,2-diacylglycerol (DAG) and inositol trisphosphate (IP3) and increases intracellular Ca^+2^ and protein kinase C (PKC). The expression of PKA and PKC promotes the transcription of melatonin-forming enzymes. Melatonin is formed in pinealocytes from tryptophan. Tryptophan is turned into hydroxy-tryptophan (5-HTP) by tryptophan hydroxylase. 5-HTP is turned into serotonin (5-HT) by decarboxylase. 5-HT is converted into N-acetylserotonin by Serotonin-N-acetyl transferase (SNAT). Finally, N-acetylserotonin is converted into melatonin by hydroxyindole-O-methyltransferase (HIOMT)

Melatonin gained its latter nickname since its synthesis and release are stimulated by darkness and diminished by light. The pineal gland acts as a neuroendocrine transducer since it manages its melatonin production based on the dark/light signal it receives from the “central biological clock”, the suprachiasmatic nucleus (SCN) in the hypothalamus [142, 143]. The SCN receives light information through the retinal-hypothalamic tract from retinal ganglionic cells [144]. Then, it sends its neural signal to the pineal gland through adrenergic sympathetic fibers after synapsing in the paraventricular nucleus (PVN) of the hypothalamus and the superior cervical ganglia (SCG) [145]. Noreadrenaline activates the pinealocyte ß adrenergic receptors, increasing cyclic adenosine monophosphate (cAMP) and protein kinase A (PKA) and α1- adrenergic receptors, increasing intracellular Ca^+2^ and protein kinase C (PKC), which promotes the transcription of melatonin-forming enzymes [146]. Pinealocytes use tryptophan as a precursor to form serotonin, passing by hydoxy-tryptopan. Serotonin is then converted into N-acetylserotonin and finally into melatonin by the enzymes Serotonin-N-acetyl transferase (SNAT) and hydroxyindole-O-methyltransferase (HIOMT), respectively [142, 143, 147]. Therefore, melatonin’s synthesis can be jeopardized by tryptophan depletion [148], especially since the pineal gland is not protected by the blood-brain barrier (BBB), so severe flactuations of plasma tryptophan levels could affect melatonin concentration [149]. Moreover, the availability of synaptic catecholamine by mono-amine oxidase (MAO) inhibitors can potentiate melatonin’s production [150]. Despite the pineal gland being the main source for melatonin, extra pineal sources share in its synthesis, such as the retina, GIT mucosa, kidney, adrenals, thyroid, pancreas, and platelets, and other brain regions including the hypothalamus, pons, medulla, cerebellum, and cerebral cortex [151]. Upon exerting its action, melatonin in the brain could be catabolized into kynurenine mtabolites, which represent the other pathway for tryptophan metabolism besides serotonin [149] (Fig. 7).

#### Receptors and mechanisms of action

**Fig. 8:**
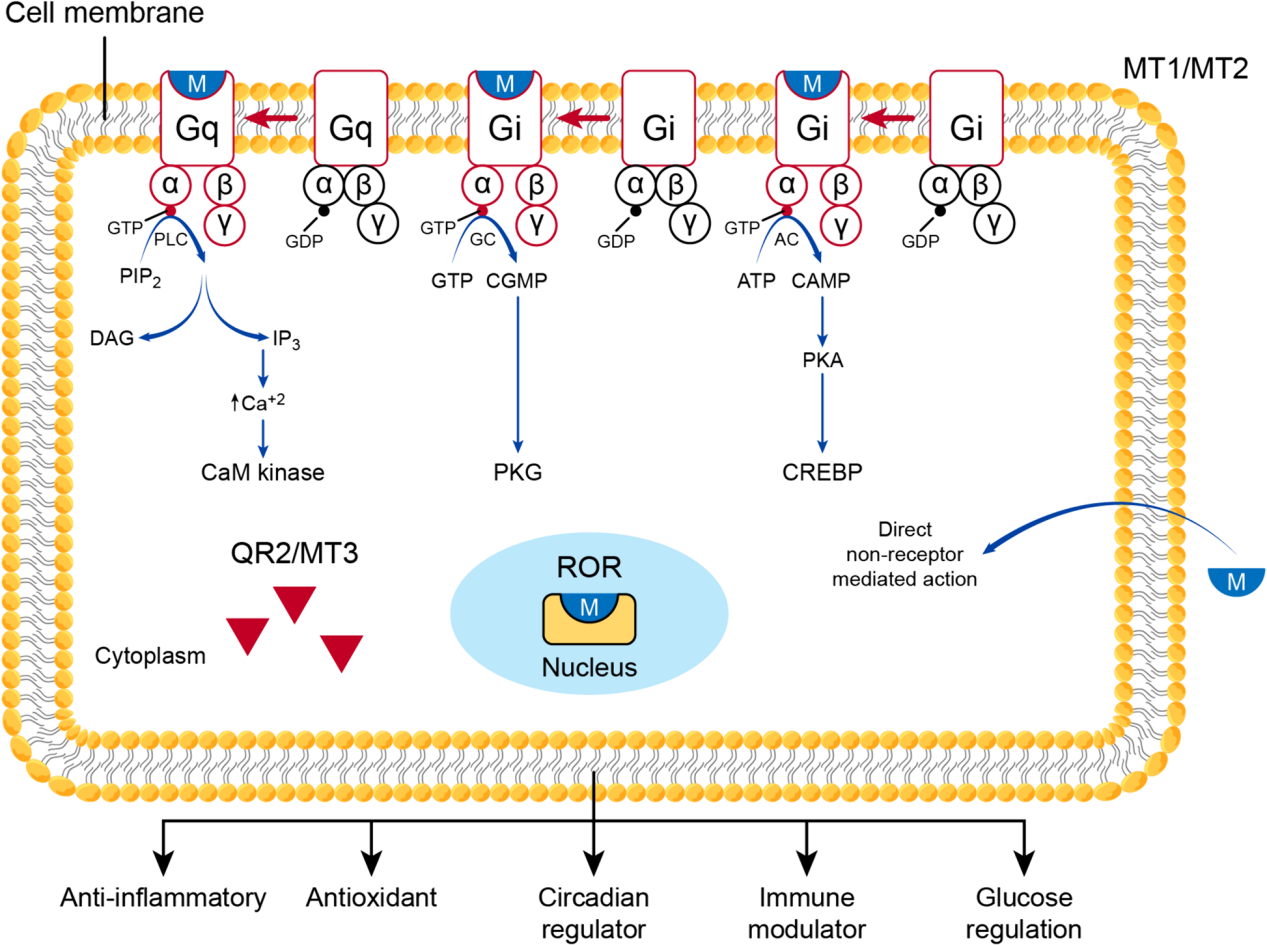
Melatonin’s receptors and mechanism of action. Melatonin can exert its various actions on cells through receptor-mediated and non-receptor mediated actions. Melatonin receptors are dispersed in the cytoplasm as MT3 receptors which are the cytosolic enzyme quinone reductase 2 (QR2), in the nucleus as the retinoid-related orphan (ROR) receptors, and on the cell membrane. On the cell membrane, melatonin acts through membrane-related G protein-coupled receptors (GPCRs) called MT1 and MT2. When melatonin binds to the Gi coupled receptors, the alpha subunit seperates from the beta and gamma subunits, with the conversion of the attached guanosine diphosphate (GDP) to guanosine triphosphate (GTP). This inhibits adenyle cyclase (AC) and the cyclic adenosine monophosphate (cAMP)/protein kinase A (PKA)/cAMP response element-binding protein (CREB) pathway. Also, the binding of melatonin to the Gi coupled receptors can inhibit the guanylate cyclase (GC)/cyclic guanosine monophosphate (cGMP)/protein kinase G (PKG) pathway. The activation of the Gq-coupled receptors activates phospholipase C (PLC), which hydrolyzes phosphatidylinositol 4,5-bisphosphate (PIP2) into inositol triphosphate (IP3) and 1,2-diacylglycerol (DAG), which increases Ca^+2^levels and activates calmodulin (CaM) and CaM kinase signaling

Melatonin can exert its plethora of functions in a receptor-mediated or non-receptor-mediated manner [152]. Owing to its lipophilic properties [153], melatonin can interact with membrane, cytosolic, and nuclear receptors [154]. Melatonin binds to two membrane-related G protein-coupled receptors (GPCRs) called MT1 and MT2 [155, 156]. GPCRs are a large family of receptors characterized by binding to guanosine di/triphosphate (GDP/GTP), hence the name. GPCRs are formed of three subunits: alpha, beta, and gamma, and based on the type of the alpha, G proteins could be Gi (inhibitory), Gs (stimulatory), Gq, or G12 [157]. MT1 and MT2 are mainly Gi coupled; therefore, melatonin’s binding inhibits adenyle cyclase and the cAMP/PKA/cAMP response element-binding protein (CREB) pathway or the guanylate cyclase (GC)/cyclic guanosine monophosphate (cGMP)/protein kinase G (PKG) pathway, respectively. However, melatonin’s receptors can be Gq-coupled and activate phospholipase C (PLC), which hydrolyzes phosphatidylinositol 4,5-bisphosphate (PIP2), producing inositol triphosphate (IP3) and 1,2-diacylglycerol (DAG), which increases Ca^+2^levels and activates calmodulin (CaM) and CaM kinase signaling [158, 159]. A third binding site for melatonin is MT3, which is the cytosolic enzyme quinone reductase 2 (QR2) [160]. QR2 is one of the reductases that help protect against oxidative stress by blunting quinones’ electron transfer reactions [161].In addition, melatonin binds to a family of nuclear receptors called the retinoid-related orphan (ROR) receptors [162], which are involved in the regulation and modulation of the circadian clock [163]. Melatonin can directly detoxify free radicals in a non-receptor-mediated fashion, either directly or through its metabolites [152] (Fig. 8).

#### Physiological roles and properties

Since being identified, melatonin’s physiological roles have witnessed multiple transitions from being known for its skin-lightening effects [134]. The anticipated dermatological role was swiftly redirected to its gonadal effects [164, 165]. The light/dark-dependent gonadal inhibitory effect of melatonin [166–168] was the spark that shined upon its unmatched physiological role as a chronobiotic hormone [169]. The fact that melatonin is a night signal shapes its entire mission of circadian rhythm regulation, especially sleep-wake rhythms and core temperature [170], along with its anti-oxidant [171], immunomodulatory, anti-inflammatory [172], and glucose regulation effects [173].

Melatonin’s production peaks at the dark phase of the day and is suppressed by light [174], with evidence of being effectively suppressed by short-wavelength blue light [175]. The secretion of melatonin represents the biological night; hence, it transduces the dark signal to the body and, as such, adjusts the timing of the central biological clock, SCN, and circadian rhythms of the body [176]. The capacity of melatonin to manipulate the body’s circadian rhythms was first witnessed on exogenous administration of 2 mg of melatonin, which advanced sleep time [177]. The circadian rhythm in the body is governed by the central clock, SCN, and an associated molecular machinery within body cells, namely the clock genes. Interestingly, melatonin is not only the handyman for the SCN, transmitting its neural signal to peripheral tissues that express melatonin receptors, but it can also feedback and manipulate both the central clock (i.e., the SCN) and the clock genes [145, 178–180]. The circadian rhythm molecular machinery depends on two major transcriptional factors: BMAL1/ARNTL (brain and muscle ARNT-like 1 or aryl hydrocarbon receptor nuclear translocator-like protein 1) and CLOCK (circadian locomotor output cycles kaput). CLOCK and BMAL1 heterodimerize and activate the transcription of circadian-controlled clock genes, including three Period (Per1, Per2, Per3) and two Cryptocrome (Cry 1, Cry 2) genes. After their translation, Per and Cry translocate to the nucleus and create a negative feedback loop, inhibiting both CLOCK and BMAL1 [181, 182]. Another route through which CLOCK and BMAL1 can function is by promoting the transcription of the nuclear receptors REV-ERBα [also known as NR1D1 (nuclear receptor subfamily 1 group D member 1)] and ROR, which is one of melatonin’s target receptors. Then, REV-ERBα and ROR form their own negative and positive feedback loop, respectively, on BMAL1 transcription [183, 184]. Melatonin evidently participates in the well-functioning of the circadian molecular machinery, and their interaction is considered one of melatonin’s substantial effects on cell survival [185].

Melatonin’s versatile actions span all body systems in a cytoprotective manner. On reproduction, melatonin decreases gonadotropin release hormone (GnRH) production [186], regulating the release of prolactin, follicle-stimulating hormone (FSH), and luteinizing hormone (LH) [187] and therefore the onset of puberty [188]. In addition, melatonin regulates ovulation, folliculogenesis, and spermatogenesis, among many other aspects of male and female reproduction [189–191]. On the skeletal system, melatonin protects bone density, promotes osteogenesis, and facilitates the differentiation of human mesenchymal stem cells (hMSCs) into bone-forming osteoblasts [192, 193]. On the cardiovascular system, melatonin regulates blood pressure circadian rhythms, exhibits an antagonistic action to angiotensin II (Ag II) [194, 195], and diminishes the flactuation of cerebral blood flow [196]. On the GIT, melatonin exerts diverse actions, including protecting the intestinal mucosa, relaxing the GIT smooth muscles, adjusting digestive enzymes, and modulating gut microbiome [197].

The CNS is specifically endowed with widely disrtributed melatonin receptors with the advantage of melatonin being directly secreted into the cerebrospinal fluid (CSF) [198]. Being available in the CSF makes melatonin’s function on neurons and glial cells more accessible, especially with their extensive expression of melatonin’s receptors [198–200]. Melatonin is an indispensible neuroprotective substance with a plethora of functions, including effects on cognition, neurotransmission, hippocampal neurogenesis, and regulation of synaptic and neural plasticity, along with its chief role as a chronobiotic [201]. Melatonin has a unique depressive effect on brain excitability. This was repeatedly demonstrated in reports of increased brain electrical activity after pinealectomy [202, 203] and diminished activation of seizures in rats receiving several epileptogenic agents upon systemic melatonin administration [204]. The uniqueness of melatonin’s action in this regard stems from its ability to modulate the two major neurotransmitters governing brain excitability. Melatonin shows a gamma aminobutyric acid (GABA)-like action, potentiates its activity on neurons [205], enhances its levels [206], and exhibits a modulating action on benzodiazepine receptors [207–209]. On the other hand, melatonin diminishes the infamous glutamate-mediated excitotoxicity. Glutamate action on N-methyl D-aspartate (NMDA) receptors, one of its ionotropic receptors, incites Ca^+2^ influx and Ca^+2^-calmodulin-dependent activation of nitric oxide synthase (NOS) which then produces Nitric oxide (NO), the signal that sustains glutamate release [210]. Melatonin can disrupt this potentially malicious cycle by binding to calmodulin, inhibiting the activation of NOS and the synthesis of NO [211–214]. To further validate the argument of melatonin’s comprehensive action on the brain, Larson et al. showed impaired long-term potentiation, the process behind long-term memory, in MT2 receptors-deficient mice, denoting an extended role of melatonin in memory processing [215]. Bechstein et al. detected worsened spatial learning in melatonin receptor-knockout (KO) mice compared to the wild type (WT). They displayed enhancement of the working memory with melatonin adminstration to melatonin-deficient C57BL/6 mice on daytime. In addition, WT mice showed an evident difference in day/night LTP, which was absent in melatonin and melatonin receptor-deficient mice, highlighting the circadian regulatory effect of melatonin on LTP [216].

### Melatonin in pathophysiological contexts

Melatonin is involved in an extensively wide arena of pathological contexts that can be explained by its multi-system action and overarching physiological properties and implications [217]. Moreover, the continuously expanding research on melatonin’s role in humans’ pathology can be partially attributed to its repeatedly-reported safety as a potential treatment [218]. Three double-blind controlled studies showed the lack of serious adverse effects of melatonin with exogenous long-term use, for more than six months. Only a few reports showed significant somnolence and mood swings, which validates the absence of concerning issues and the reasonable tolerability of melatonin use [219]. Nonetheless, the use of melatonin needs cautionary measures when used with other medications, especially anticoagulants such as warfarin, since their interaction could increase the risk of bleeding [220–222] To show the variability of its pathological involvement, melatonin is implicated in sleep disorders [223, 224], precocious puberty [225], polycystic ovary syndrome, endometriosis [226], coronary heart diseases [227], GIT disorders [228], cancer [229], diabetes [230], obesity and energy metabolism issues [231], and a wide array of psychiatric and neurological disorders [232]. The proposed therapeutic efficacy of melatonin for the post-COVID-19 trajectory of brain aging and neurodegeneration is based upon melatonin’s long history with aging, neurodegenerative disorders, viruses, and most recently, SARS-CoV-2 infection.

#### Melatonin and aging, neurodegenerative disorders, and viruses

Aging has been repeatedly claimed to be a secondary event to pineal malfunction, with an apparent loss of melatonin’s day-night rhythm and a drop in the magnitude of its nighttime production [233–236]. Melatonin has anti-aging properties and is extensively reported to combat age-related disorders on both experimental and clinical levels. Twenty-two-months-old rats fed with melatonin for ten weeks showed better bone structure and diminished age-related bone loss [237]. An interesting study found a 42% prolonged life span in old mice when subjected to pineal gland transplantation from young ones and a 29% reduction in life span with pineal transplantation from the old to the young ones [238]. In addition, melatonin improved immune functions in aged immune-compromised mice [239]. In a d-galactose-induced aging model, melatonin enhanced the associated spatial memory defects and regained neuronal proliferation [240]. In senescence-accelerated prone mice (SAMP8), melatonin exhibited improvement in hippocampal neurogenesis [241]. Melatonin favors anti-aging outcomes through its anti-inflammatory properties, suppressing the process of inflammaging [242]. In addition to its myriad of beneficial qualities, melatonin showed an anti-oxidant effect in the hippocampus of aged rats [243]. In a randomized controlled study, melatonin improved sleep quality and morning alertness in aging patients with primary insomnia [244]. Melatonin improved post-operative cognitive decline, fatigue, and general well-being in elderly patients with hip arthroplasty [245]. A retrospective study on patients with mild cognitive impairment (*n* = 25) on 9-18 months of melatonin (3-9 mg) showed better performance in the Mini Mental State Examination (MMSE), the cognitive subscale of the Alzheimer’s Disease Assessment Scale, and multiple neuropsychological tests [246].

Being referred to as age-related disorders, multiple studies on neurodegenerative disorders displayed a positive outcome with melatonin. Melatonin showed a recurrent theme of improvement of cognitive deficits, memory performance, and associated anxiety and depression in various mouse models of AD [247–249]. Melatonin projected the same positive image in experimental models of PD, improving neurobehavioral tasks and neural deficits in a cytoprotective manner [250–252]. In addition, in experimental models, melatonin ameliorated the severity of multiple sclerosis (MS) [253] and improved survival time in amyotrophic lateral sclerosis (ALS) [254]. Luckily, these results are, to some extent, mirrored in clinical studies. A meta-analysis of 50 randomized controlled trials in AD patients concluded a better score on the MMSE ( the medium-term low-dose melatonin to be associated with the highest post-treatment MMSE (mean difference = 1.48, 95% CI 0.51-2.46), assessing cognitive functions, with medium-term low-dose melatonin [255]. Low melatonin levels have been linked to cognitive decline [256] and trough, but not daytime [257]. Melatonin levels were associated with cognitive dysfunction as measured by MMSE in patients with mild cognitive impairment or Alzheimer’s dementia [258]. Obayashi et al. reported that higher melatonin metabolite concentrations (urinary 6-sulfatoxymelatonin: UME) are associated with significantly lower cognitive performance in the MMSE (*n* = 935) and in the Geriatric Depression Scale (*n* = 1,097) [259]. In a randomized controlled clinical trial in 60 PD patients, 12 weeks of melatonin administration (10 mg/d) improved cognition, anxiety, and depression compared to patients on placebo [260]. A pilot study of fourteen patients with MS on 6 mg of melatonin at bedtime exhibited better physical and cognitive tasks, including posturographic tests, Montreal cognitive assessment, and simple reaction time test. They also reported better sleep quality on the Spiegel’s sleep questionnaire [261]. A retrospective analysis of the Pooled Resource Open-Access Clinical Trials (PRO-ACT) database demonstrated a declining death rate in melatonin users compared to non-users among ALS patients (95% CI 0.088–0.659, *P* = 0.0056). Interestingly, melatonin users also had a slower decline in the Revised Amyotrophic Lateral Sclerosis Functional Rating Scale (ALSFRS-R) (t = 2.71, *P* = 0.0069), which assesses patients’ physical functions and daily activity [262].

As broadly implicated in aging and neurodegenerative disorders, melatonin has its own relevance in viral infections, portraying its magnificent versatility. The encompassing anti-inflammatory, anti-oxidant, anti-apoptotic, and immune modulatory effects of melatonin enable it to easily behave as an efficient anti-viral [263]. Melatonin came to the rescue in previous viral outbreaks, such as the Venezuelan equine encephalomyelitis (VEE) infection in 1995 [264], West Nile encephalitis in 2002 [265], the severe acute respiratory syndrome (SARS) in 2003 [266], and the Ebola virus in 2013 [267]. The highlights of melatonin’s anti-viral action are that it reduces viremia, viral load in the brain, encephalitis, acute lung injury, and mortality rates [263].

#### Melatonin use in COVID-19

Once again, with the COVID-19 pandemic, heads turned towards melatonin and its therapeutic potential, especially with its deficient levels in SARS-CoV-2-positive patients. Yilmaz and Oner found lower plasma melatonin levels in COVID-19-positive pneumonia patients compared to healthy controls at 23:00 h and compared to the COVID-19-negative pneumonia group and healthy controls at 02:00 h and 06:00 h. Interestingly, they reported a negative correlation between the plasma level of melatonin and scores of the Beck Depression Inventory (BDI), Templer Death Anxiety Scale (TDAS), and Insomnia Severity Index (ISI) in SARS-CoV-2-positive patients [268]. Their findings imply a potential role of melatonin deficiency in COVID-19-associated depression, anxiety, and insomnia, which encourages further research to deploy melatonin in COVID-19 patients with such complications. Melatonin deficiency in COVID-19 can arise from the faulty absorption of dietary tryptophan because SARS-CoV-2 downregulates angiotensin-converting enzyme-2, the chaperone of the tryptophan transporter [269]. Several attempts have been made to investigate the impact of melaotonin on the COVID-19 infection course. In a study of 44 COVID-19 cases, Farnoosh et al. reported decreased pulmonary involvement, fatigue, CRP levels, time to discharge, and a more prompt return to baseline health in patients receiving melatonin at a dose of 3 mg three times daily for 14 days [270]. Another pilot study by Fogleman et al. reached a similar outcome in patients with mild to moderate COVID-19 symptoms receiving melatonin 10 mg/d for 14 days, showing quicker symptoms resolution [271]. A larger randomized controlled trial with 118 ICU-admitted severe COVID-19 cases used 5 mg of melatonin twice daily for 7 days. Interestingly, they depicted a significant improvement in the mortality rate, the need for invasive mechanical ventilation, the hospital and ICU length of stay, and the duration until clinical improvement in the melatonin group. Also, they found a significant improvement in CRP, erythrocyte sedimentation rate (ESR), procalcitonine, D-dimer, and serum ferritin levels during the duration of the intervention with melatonin [272]. Besides the improved hospital course, melatonin ameliorated the frequency of some of the COVID-19 ruthless complications. Hasan et al. monitored the occurrence of sepsis and thrombosis among 158 severe COVID-19 patients, of whom 82 received 10 mg/d melatonin for 14 days along with standard therapy. Patients on melatonin were less likely to develop thrombosis and sepsis and had a lower mortality rate [273]. Nevertheless, some reports renounced melatonin’s potential for a better COVID-19 course and outcome. In a pilot randomized trial of 21 severe COVID-19 cases, Darban et al. reported no difference in the oxygen saturation, inflammatory markers, or ICU length of stay between patients on melatonin, zinc, and vitamin C added to the standard treatment and those on standard care only [274]. Similarly, in a retrospective observational study, Sánchez-Rico et al. demonstrated no impact of melatonin on the mortality rate among hospitalized COVID-19 patients [275]. Sahu et al., another retrospective study, viewed the impact of melatonin at the opposite end of the spectrum, showing a longer length of hospital stay among patients on 6 and 9 mg/d of melatonin and an absent positive impact on mortality rates [276]. A better approach to resolving the discrepancy of melatonin’s effect would by performing well-designed controlled studies with larger sample sizes [277]. Despite the contradictory clinical reports, animal studies support melatonin’s positive impact. In a K18-hACE2 mouse model of COVID-19, 2 days of melatonin pre-infection and 7 days post-infection with SARS-CoV2 minimized the brain viral load, small vessel damage, and brain inflammation [278]. Additionally, Yadalam et al. demonstrated, in an in silico study, a significant intermolecular interaction of melatonin and its receptor agonists, agomelatine and ramelteon, with the receptor binding domain of the SARS-CoV-2 spike protein and the ACE2 receptors which makes the notion of melatonin hindering the SARS-CoV-2 cellular entry more credible [279]. In the same light, using melatonin and its receptor agonists before and after SARS-CoV-2 infection in K18-hACE2 mice brought about better survival rates and higher clinical scores. However, there were no changes in the plasma cytokine panel or lung inflammatory markers except for higher expression of type I and type III interferon (IFN) in the lungs with melatonin treatment [280]. Despite the undeniable data on how useful melatonin could be during the course of COVID-19 infection, the use of melatonin against post-COVID sequelae is still an unexplored territory on both clinical and experimental levels. What can boost melatonin’s chances is its history against multiple viral infections as a free radical scavenger, an anti-inflammatory, and an anti-apoptotic molecule [263], as well as the intriguing intersection it has with the potential post-COVID-19 pathogenesis and ferroptosis.

### Melatonin as a ferroptosis inhibitor in the post-COVID-19 trajectory of aging and neurodegeneration: a mechanistic hypothesis

#### Ferroptosis in the post-COVID-19 trajectory of aging and neurodegeneration

**Fig. 9:**
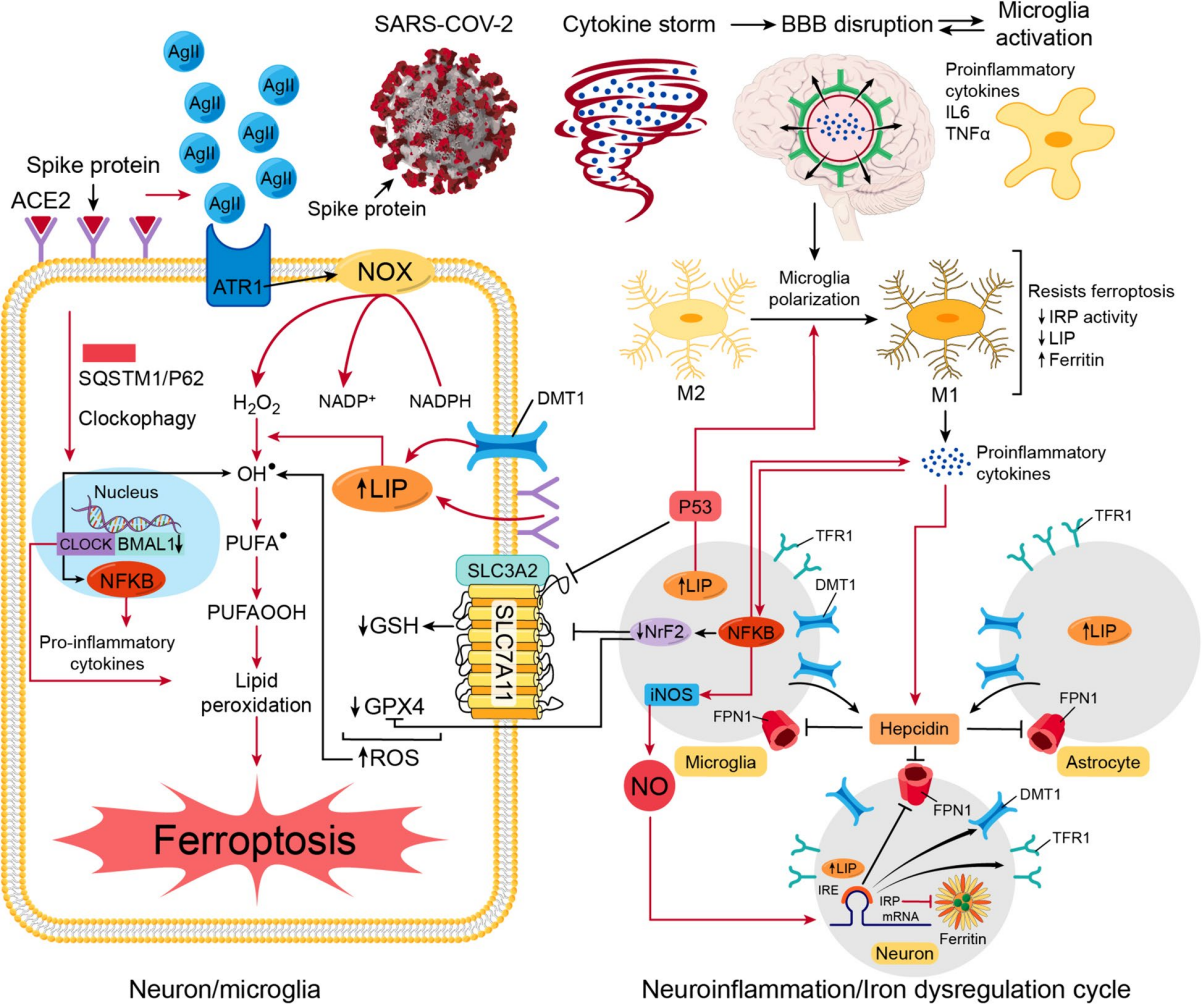
Ferroptosis as a hyothetical mechanism for the post-COVID-19 trajectory of aging and neurodegeneration. In COVID-19, five shared events can prelude to ferroptosis in post-COVID-19 trajectory. They are 1-Neuroinflammation, 2-Iron dysregulation, 3-Reactive oxygen species (ROS)/antioxidant imbalance, 4- ACE2/Ag II disruption, and 5-Clock gene alteration. 1- The SARS-CoV-2-induced cytokine storm disrupts BBB, which allows pro-inflammatory cytokines to pass to the brain vicinity and activate microglia. Pro-inflammatory ctokines aid the microglia polarization from M2 (anti-inflammatory) to M1 (Pro-inflammatory). 2- IL-6 promotes the production of hepcidin from microglia and astrocytes. Hepcidin degrades FPN1, which traps iron in neurons, microglia, and propably astrocytes. In iron-overloaded microglia, higher LIP causes more microglia polarization and NF-κB helps production of more pro-inflammatory cytokines and activates the iNOS which increases the production of NO. NO passes to neurons and modulates the molecular machinery of iron regulation by upregulating IRP, which causes higher expression of DMT1 and TFR1 and lower expression of ferritin and FPN1. This disruption in iron-related proteins increases the LIP and incites a vicious cycle of iron dysregulation and neuroinflammation. 3- The higher expression of NF-κB decreases the expression of NrF2. The iron overload activates P53. The less NrF2 and more P53 repress the expression of SLC7A11, which decreases the production of GSH. The less NrF2 also can decrease the production of GPX4, compromising the antioxidant system in the face of higher H2O2 and OH^●^ production caused by NOX and high LIP, respectively. 4- The binding of the spike protein to ACE2 receptors degrades them and Ag II accumulates and binds to ATR1, activating NOX and producing more H2O2. 5- SARS-CoV-2 could induce autophagic degradation/clockophagy of BMAL1 and CLOCK, which is mediated by the cargo receptor SQSTM1/p62. The high LIP, the disruption of ACE2/ATR1, the antioxidant system failure, and BMAL1-CLOCK deficiency all would promote lipid peroxidation and ferroptosis in a progressive manner that would lead to acceleration of aging and neurodegeneration. ROS: reactive oxygen species, ACE2: angiotensin converting enzyme 2 receptors, Ag II: angiotensin II, SARS-CoV-2: severe acute respiratory syndrome coronavirus 2, BBB: blood brain barrier, IL-6: interleukin-6, TNF-α: tumor necrosis factor alphs, FPN1: ferroportin, LIP: labile iron pool, NF-κB: nuclear factor-κB, iNOS: inducible nitric oxide synthase, NO: nitric oxide, IRP: iron regulatory protein, IRE: iron responsive element, mRNA: messenger ribonucleic acid, DMT1: divalent metal transporter 1, TFR1: transferrin receptors-1, NrF2: nuclear factor erythroid 2-related factor 2, P53: tumor protein p53, SLC7A11: Solute carrier family 7 member 11, SLC3A2: Solute carrier family 3 member 2, GSH: reduced glutathione, GPX4: glutathione peroxidase 4, H202: hydrogen peroxide, OH^●^: hydroxyl radical, PUFA^●^: polyunsaturated fatty acid lipid radical, PUFAOOH: lipid hydroperoxide radical, NOX: nicotinamide adenine dinucleotide phosphate oxidase, NADPH: nicotinamide adenine dinucleotide phosphate, ATR1: angiotensin receptor 1, BMAL1: brain and muscle ARNT-like 1 or aryl hydrocarbon receptor nuclear translocator-like protein 1, CLOCK: circadian locomotor output cycles kaput, SQSTM1/p62: sequestosome1

To view melatonin’s potential anti-ferroptosis effect, we need to first display how COVID-19 and its trajectory intersect with ferroptosis. We propose an intricate intersection between five major events that will ease the way for ferroptosis to occur, including neuroinflammation, iron dysregulation, GPX4/GSH/SLC7A11, antioxidant defenses, repression, ACE2 receptors-degradation-dependent events, and finally clock genes disruption (Fig. 9).

In COVID-19 patients, altered iron metabolism, depletion of GSH [281], inactivation of GPX4 [282], and up-regulation of lipid peroxidation biomarkers strongly propose ferroptosis as a plausible mechanism for COVID-19 multi-organ affection, including neuropsychiatric sequelae [283]. In an ischemic stroke model, ACSL4 promoted ferroptosis-induced brain injury and neuroinflammation with similar findings to neuro-COVID-19 events, such as infarct size increase, reduced neurological function, microglial activation, and increased pro-inflammatory cytokines (tumor necrosis factor alpha (TNF-α), IL-6, and IL-1β) [284]. In the context of COVID 19, we can see strong indicators of iron perturbation along with neuroinflammation in several forms. High serum ferritin [285] and hepcidin levels [286], low serum iron levels [287], and low transferrin saturation [288] have been significantly correlated with COVID-19 severity, hospitalization, and mortality [289–291].

The cytokine storm is a prominent feature of the SARS-CoV-2 infection, instigating systemic flooding with pro-inflammatory cytokines such as IL-6, IL-1β, IL-8, IFN-γ, and TNF-α [292, 293]. Moreover, the cytokine storm co-exists with a massive increase in coagulopathies and acute phase reactants such as C-reactive protein (CRP) and serum ferritin, which correlate with the severity of the disease [294–296]. High levels of peripheral pro-inflammatory cytokines compromise the BBB integrity, cross over to the brain vicinity, and activate its resident immune cells, causing microglial activation, which in turn creates a medium of neuroinflammation [297].

Even out of the context of COVID-19, inflammation has been tightly intertwined with altered iron homeostasis [298]. Conditions of infection and inflammation, specifically high levels of IL-6, tend to increase the expression of hepcidin, which then degrades FPN1. This strategy helps iron sequestration from microorganisms by decreasing iron systemic availability [299–302]. However, iron hoarding in cells could have massive downsides. In the brain, a bidirectional relationship between iron overload and neuroinflammation creates a vicious cycle that could eventually boil down to ferroptosis-mediated neuronal death. In cases of neuroinflammation, pro-inflammatory cytokines (e.g., IL-6, TNF-α) are reported to increase microglial and astrocytic hepcidin expression, which in turn degrades FPN1 in both cells and in neurons. Also, pro-inflammatory cytokines promote the overexpression of DMT1, which allows the importation of non-transferrin-bound iron in all three cells. However, the manipulation of iron transporters through pro-inflammatory cytokines seems to impact only neurons and microglia, resulting in cellular iron overload in these specific cells [303–305]. Pro-inflammatory cytokines and iron overload activate microglia and increase its polarization from the anti-inflammatory phenotype (M2) to the pro-inflammatory phenotype (M1), which aggravates pro-inflammatory cytokine production, including NO [306]. Iron and pro-inflammatory cytokines activate the transcriptional factor nuclear factor-κB (NF-κB), which enhances the production of inducible nitric oxide synthase (iNOS), converting arginase into NO [307, 308]. Then, NO from the activated microglia gets to reshape neuronal iron homeostasis by targeting the IRE/IRP system [298, 309]. NO activates IRP1, which halts the translation of FPN1 and ferritin and promotes that of DMT1 and TfR1, favoring the increment of the cellular labile iron pool, which triggers the Fenton reaction and ferroptosis-mediated neuronal cell death [80, 310, 311]. However, the microglia tend to behave differently regarding handling iron according to their phenotype, which impacts their sensitivity to ferroptosis. M1 microglia are more resistant to ferroptosis than M2 microglia since they have less IRP activity and cellular labile iron pool, and higher ferritin levels, which help maintain neuroinflammation, perpetuate iron dysregulation, and ferroptosis-mediated neuronal death [298, 310, 312].

Aging, including brain aging, is an intricate process with a multitude of underlying events such as inflammation, oxidative stress, metabolic derangement, mitochondrial dysfunction, DNA damage, and immunosenescence, in which cells attain SASP by releasing cytokines, chemokines, growth factors, and reactive metabolites [313–315]. This multi-faceted process of aging seems to perfectly align with much of the COVID-19 pathophysiology [316, 317]. Progressive, persistent systemic inflammation with an ongoing rise in pro-inflammatory cytokines such as IL-6, IL-1β, TNF-α, and iNOS holds its position as a consequence of COVID-19 infection [318] and as a prelude to aging, a process termed inflammaging [315]. In a retrospective cohort of 272 of hospitalized SARS-CoV-2-confirmed patients, serum inflammatory cytokines such as IL-2R (receptors) and IL-6 were detected in older patients (≥ 65 years). IL-2R, IL-6, IL-8, and IL-10 were significantly higher in severely ill patients from week 1 to week 5 post infection [319]. Also, the CSF IL-6, IL-8, IL-15, and monocyte inhibitory protein (MIP)-1β levels were higher in COVID-19 patients with neurological symptoms compared to controls and human immunodeficiency virus (HIV) patients, with evidence of BBB disruption [320]. Another tipping point where COVID-19 and aging seem to meet is iron dysregulation. During the process of aging, the systemic availability of iron tends to decline with higher levels of intracellular iron, posing the threat of inciting ferroptosis. The intracellular trapping of iron arises from the downregulation of FPN1 with high hepcidin levels due to aging-associated chronic inflammation [321–326]. Iron dysregulation is strongly associated with a premature phenotype of aging termed ferrosenescence, which is triggered by intracellular iron retention. This type of aging is characterized by DNA damage, telomere shortening, and methylation defects [327], which were reported in COVID-19 patients [113]. Telomere dysfunction seems to activate p53 cellular responses [328]. P53 is a key regulator of cells’ DNA repair mechanisms; however, viral infection-associated inflammatory responses could lead to the persistent activation of p53 [317]. In addition, iron overload tends to activate p53 acetylation and mediate M1 macrophage polarization in a p53-dependent manner, sustaining pro-inflammatory responses [329]. Also, NCOA4-mediated ferritin degradation was reported in the brains of aging mice [330].

The massive cytokine storm associated with COVID-19 infection creates neuroinflammation, resembling one of the key events that fosters neurodegeneration [331]. AD brains reflect high levels of pro-inflammatory cytokines, including IL-6, IL-1β, and TNF-α secreted by activated microglia [332, 333]. Iron dysregulation is closely related to neurodegeneration. Brain iron is significantly elevated in different cortical regions in AD brain autopsies, with strong association between brain iron content and cognitive decline [334]. Iron exists within neurofibrillary tangles and accelerates tau phosphorylation [335]. Bao et al. reported the downregulation of FPN1, the only iron cellular exporter, in the hippocampus of the APPswe/PS1dE9 AD mouse model and in AD patients brain tissues. They developed a conditional FPN1 KO mouse model where the FPN1 is deleted in the principal neurons in the hippocampus and neocortex. The FPN1 deficiency resulted in cognitive impairment and an evident increase in ferroptosis markers. Also, the use of ferroptosis inhibitors ameliorated the Aβ-induced neuronal cell death and memory deficits [101]. Ferroptosis has emerged as a plausible culprit for AD pathology. In AD, iron overload activates microglia, which maintains neuroinflammation and AD-related protein aggregation. Iron activates NF-κB in microglia which triggers the production of pro-inflammatory cytokines, including NO. NO can render microglia resistant to ferroptosis, which allows its continuous inflammatory response, inflicting harm on neurons and promoting their ferroptosis-mediated death [310].

Besides the maliciously vicious cycle of iron dysregulation and neuroinflammation, SARS-CoV-2 renders cells vulnerable to ROS and lipid peroxidation through the collapse of antioxidant defenses and inhibition of the GPX4/GSH machinery. Also, SLC7A11 seems to be involved in the potential SARS-CoV-2- mediated ferroptosis [336–338]. P53 activation has been reported as an important player in the context of ferroptosis-related aging. P53 activation mediates ferroptosis by repressing the transcription of SLC7A11 or system Xc-, resulting in cysteine deficiency, GSH depletion, and ferroptosis [339, 340]. More interestingly, a higher labile iron pool with ferritin degradation was reported to enhance p53/SLC7A11-mediated ferroptosis [341]. Iron overload-triggered expression of p53 was reported to promote SLC7A11-repression mediated ferroptosis in aging neurons and in muscles [342, 343]. The transcription factor NrF2 stands as an opponent for SLC711-p53-mediated repression since it promotes the expression of SLC7A11 as one of its target genes, which opposes ferroptosis [344]. In addition, NrF2 increases the expression of H ferritin, which assists the intracellular storage of iron as ferritin and decreases the labile iron pool [105], a noble role that alleviates iron-related brain aging [345], and its absence could foster premature aging [346]. Keeping up with the notion of aligned events in aging and COVID-19 infection, suppressed NrF2-dependent gene expression was reported in COVID-19 patients, and NrF2 agonists halted SARS-CoV-2 replication [347]. Moreover, p53 activation is involved in SARS-CoV-2 spike protein-mediated neurotoxicity [348]. Higher expression of p53 and p21, an aging marker, mediated the senescence of retinal pigment epithelial cells treated with SARS-CoV-2 spike protein [349]. Neurodegeneration in COVID-19 patients is a potential consequence of GSH depletion and GPX4 inactivation [350], which would promote lipid peroxidation and ferroptosis [283]. GSH synthesis in AD is disrupted and associated with worsened pathology. Thioredoxin compromises an important step in the formation of GSH and was found reduced in AD brains [351]. Chiang et al. reported lower total glutathione levels in brain regions in AD patients with amyloid plaque deposition [352]. In a 5xFAD, five familial AD-related mutations, AD mouse model, mice exhibited markers for lipid peroxidation and ferroptosis along with cognitive decline, which improved with the generation of transgenic 5xFAD mice that overexpress GPX4 [353], displaying the crucial role of GPX4 in the mediation of ferroptosis-induced neurodegeneration.

Another possible route for ferroptosis is direct viral neuropathology through the ACE-2 receptors. SARS-CoV-2 binds to ACE-2 receptors to enter cells, degrade them, and in turn increase Ag II which tends to act on angiotensin receptor 1 (ATR1). The activation of ATR1 would activate NOX enzymes, which promote the production of hydrogen peroxide, lipid peroxidation, and ferroptosis [337]. Moreover, NOX is reported to increase microglial polarization towards the pro-inflammatory M1 phenotype, which would help maintain a state of neuroinflammation [354]. On the other hand, the activation of ACE2 receptors limits activated microglia polarization to the M1 phenotype and shifts its polarization to the M2 anti-inflammatory phenotype [355], which demonstrates the critical role of the renin-angiotensin system (RAS) in neuroinflammation. RAS is another point where aging, ferroptosis, and COVID-19 meet [337], since RAS has been repeatedly implicated in aging. The activation of ATR1 was reported to activate p53/p21 and cause vascular senescence [356]. Ag II seems to induce vascular ferroptosis through activation of p53 [357]. ATR1/NOX activation promotes oxidative stress, neuroinflammation, age-related changes, and even iron-induced microglial pro-inflammatory polarization [354, 358, 359]. Ag II modulates iron homeostasis in aging, where it shows hyperactivity, associated oxidative stress, an inflammatory response, and increased microglial iron deposition. Also, chronic administration of Ag II resulted in lower levels of ferritin and an increased labile iron pool [360]. Ag II accelerates cellular senescence by causing DNA damage and telomere shortening [361]. Activation of ACE2 receptors and blocking ATR1 has been a recurrent theme in the context of AD to ameliorate cognitive functions, decrease brain pro-inflammatory cytokines, Aβ, and hyperphosphorylated tau burden [362–365]. Moreover, brain-derived neurotrophic factor (BDNF), which is a crucial player for neurodevelopment and cognitive functions, showed lower levels in ACE2KO mice, with higher ATR1 expression and cognitive deficits [366].

The altered expression of clock genes stands at the crossroads of ferroptosis, COVID-19, aging, and neurodegeneration. The circadian clock has been repeatedly implicated in viral replication, associated pathogenesis, and host responses, and SARS-CoV-2 is no exception. ACE2 receptor expression is controlled by the two circadian transcriptional factors, BMAL1/ARNTL and CLOCK [367–369]. Zhuang et al. demonstrated BMAL1/ARNTL-controlled SARS-CoV-2 cell entry and replication [370], manifesting a strong link between SARS-CoV-2 and clock genes. Moreover, variation in CLOCK and BMAL1/ARNTL expression in monocytes impacts cytokine production [371], showing the effect clock genes could have on COVID-19 progression. On the other hand, SARS-CoV-2 could alter the function of BMAL1/ARNTL and possibly other clock genes [367, 372]. BMAL1/ARNTL deficiency triggers the production of pro-inflammatory cytokines [373], which could pave the way for further consideration of BMAL1/ARNTL in the extended pathogenesis of COVID-19. A form of autophagic degradation of clock genes called clockophagy is reported to have a crucial role in ferroptosis. The autophagic degradation of BMAL1/ARNTL mediated by the cargo receptor SQSTM1/p62 (sequestosome1) is reported to promote lipid peroxidation and ferroptosis [106, 374]. SQSTM1/p62 has been reported as a major player in the process of aging, with contradictory effects [375, 376]. Intriguingly, iron and clock genes happen to intersect with aging. Liu et al. reported a plausible and crucial role for iron accumulation in the altered circadian rhythm and clock gene expression associated with aging [377]. Clock gene disruption is a frequent finding with aging [378, 379]. BMAL1-deficient mice showed premature aging, higher production of ROS, and reduced life spans [380]. Also, Ali et al. reported accelerated aging with higher expression of the cell cycle inhibitor p21 and cognitive deficits in BMAL1-deficient mice [381]. All this suggests clock genes, especially BMAL1, as a strong candidate in the pathogenesis of the possible COVID-19-induced acceleration of aging, with a potential role for iron disruption and ferroptosis.

The circadian clock genes are implicated in neurodegeneration [382] as well as the pathogenesis of COVID-19 [367]. 1-methyl-4-phenyl-1,2,4,5-tetrahydropyridine (MPTP)-treated Parkinson’s mouse model showed inactivation of the main circadian transcriptional factor, BMAL1/ARNTL, resulting in motor dysfunction, dopaminergic neuron loss, and aggravated activated microglia-mediated neuroinflammation, displaying the role of BMAL1/ARNTL in microglial pro-inflammatory responses [383]. In an AD mouse model, cognitive decline was associated with BMAL1/ARNTL deficiency, which promoted microglial-derived chronic inflammation through NF-κB activation [384]. The autophagic degradation, or clockophagy, of BMAL1/ARNTL1 promotes ferroptosis [374, 385]. This alludes to the hypothesis that COVID-19-mediated dysfunctional activity and degradation of BMAL1/ARNTL can mediate chronic microglial-inflammatory responses and neuronal ferroptosis-mediated demise.

#### Melatonin as a ferroptosis inhibitor in the post-COVID-19 trajectory of aging and neurodegeneration

**Fig. 10:**
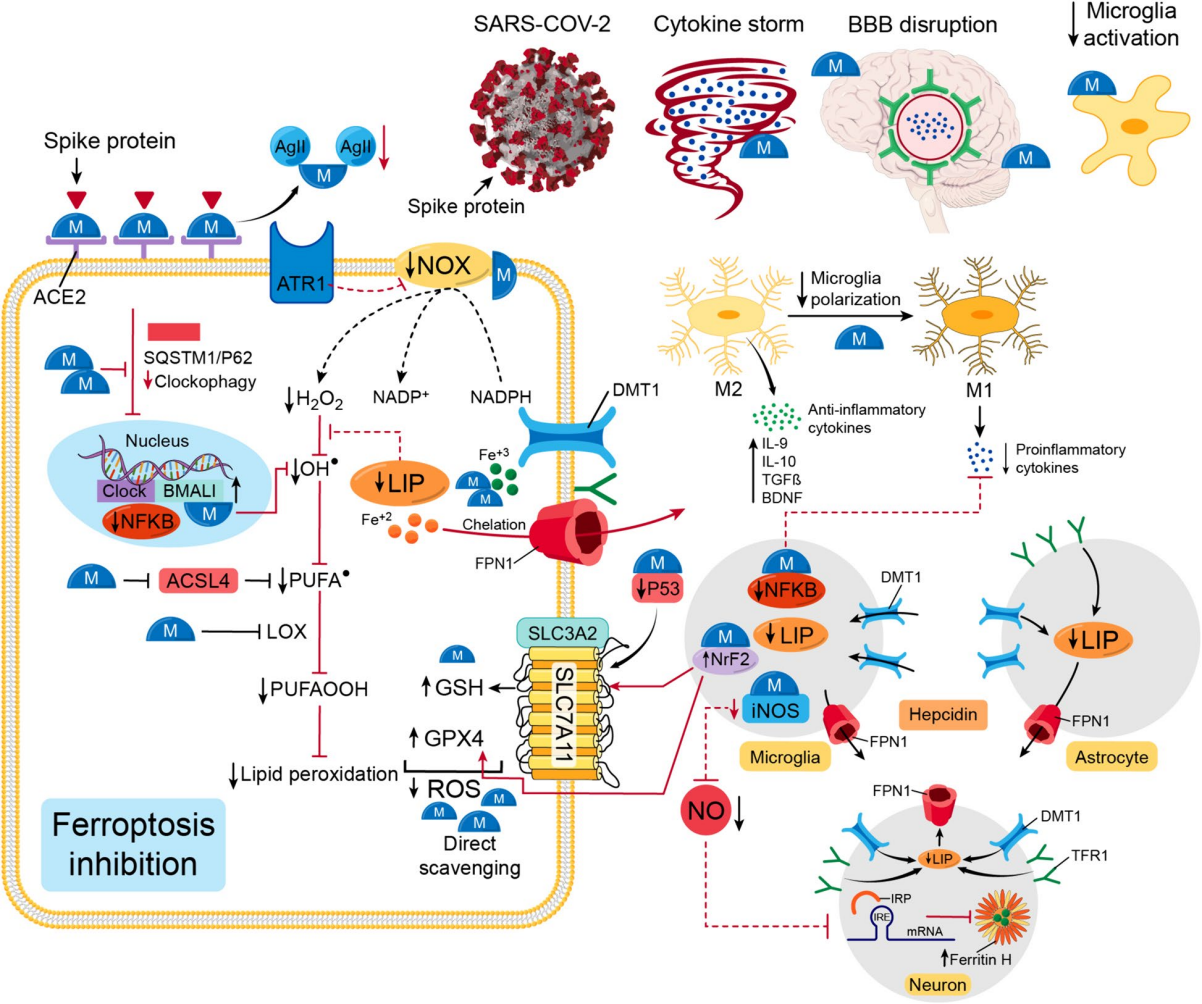
Melatonin as a ferroptosis inhibitor in the post-COVID-19 trajectory of aging and neurodegeneration. Melatonin as an anti-inflammatory can ease the impact of the SARS-COV-2-induced cytokine storm, which would decrease the chances of BBB disruption and passage of pro-inflammatory cytokines to the brain vicinity. Melatonin can reduce the microglial activation and polarization from M2 (anti-inflammatory) to M1 (pro-inflammatory), which increases the production of BDNF, IL-9, IL-10, and TGF-β. The lower incidencce of neuroinflammation would decrease the chances of iron dysregulation. Melatonin decreses the expression of NF-κB, which decreases the production of anti-inflammatory cytokines and NO, protecting against its harmful impact on iron molecular regulatory machinery (IRE/IRP). Melatonin also can act as iron chelator, diminishing the iron overload/neuroinflammation cycle. Melatonin decreases can activate the SLC7A11 by activating the NrF2 and depression of P53, which would accommodate production of GSH. Also, melatonin acts as an antioxidant by direct scavenging of ROS, helping production of GSH and GPX4, and iron chelation. Melatonin can interfere with the binding of spike protein to ACE2 and can act as an antagonist against Ag II, which would decrease the activation of NOX and production of H2O2. Melatonin can inhibit the process of clockophagy and prevent the deficiency of BMAL1 and CLOCK, decreasing ROS production, lipid peroxidation, and pro-inflammatory cytokines production. Melatonin can reduce lipid peroxidation by inhibiting ACSL4 and LOX, which would decrease the PUFA incorporation in cell membranes and the production of PUFAOOH. The integrated action of melatonin can perfectly protect against ferroptosis. SARS-CoV-2: severe acute respiratory syndrome coronavirus 2, BBB: blood brain barrier, BDNF: brain derived neurotrophic factor, IL-9: interleukin 9, IL-10: interleukin 10, TGF-β: tumor growth factor- β, NF-κB: nuclear factor-κB, FPN1: ferroportin, LIP: labile iron pool, iNOS: inducible nitric oxide synthase, NO: nitric oxide, IRP: iron regulatory protein, IRE: iron responsive element, mRNA: messenger ribonucleic acid, DMT1: divalent metal transporter 1, TFR1: transferrin receptors-1, Fe^+2^: ferrous iron, Fe^+3^: Ferric iron, NrF2: nuclear factor erythroid 2-related factor 2, P53: tumor protein p53, SLC7A11: Solute carrier family 7 member 11, SLC3A2: Solute carrier family 3 member 2, GSH: reduced glutathione, GPX4: glutathione peroxidase 4, H202: hydrogen peroxide, OH^●^: hydroxyl radical, PUFA^●^: polyunsaturated fatty acid lipid radical, PUFAOOH: lipid hydroperoxide radical, NOX: nicotinamide adenine dinucleotide phosphate oxidase, NADPH: nicotinamide adenine dinucleotide phosphate, ROS: reactive oxygen species, ACE2: angiotensin converting enzyme 2 receptors, Ag II: angiotensin II, ATR1: angiotensin receptor 1, BMAL1: brain and muscle ARNT-like 1 or aryl hydrocarbon receptor nuclear translocator-like protein 1, CLOCK: circadian locomotor output cycles kaput, SQSTM1/p62: sequestosome1. ACSL4: synthetase long-chain family member 4

Melatonin has been previously introduced as a ferroptosis inhibitor in various contexts; however, we reintroduce melatonin in a new light as a plausible protagonist, mitigating the post-COVID-19 trajectory of aging and neurodegeneration. By tracking the previously proposed hypothesis, we find melatonin brings the answer in every step (Fig. 10).

Melatonin can mediate its anti-ferroptotic effect by being an efficient anti-inflammatory. Melatonin counteracts inflammation by inhibiting microglail activation and its polarization to the proinflammatory M1 phenotype [386, 387]. Melatonin can foster the production of anti-inflammatory cytokines such as IL-10, IL-19, and tumor growth factor (TGF)-β, along with increased expression of BDNF in microglia [388]. In cellular and mouse PD models, melatonin decreased the polarization of microglia to M1 and increased the anti-inflammatory M2 through upregulation of its RORα receptors that were downregulated with PD induction [389]. In a D-galactose model of accelerated aging, melatonin improved memory through inhibition of microgliosis and downregulation of NF-κB and NOS [390]. Melatonin inhibits the activity of iNOS and the production of NO [213], which stands behind the anti-ferroptosis action of melatonin in intervertebral disc degeneration [391]. The reduction of iNOS can abolish its ferroptosis resistence effect on M1 microglia, cutting the continuum of the state of neuroinflammation [392].

Melatonin does not stop at being an anti-inflammatory but rather modulates iron homeostasis and works as an iron chelator with a ferrous iron (Fe^+2^) chelating capacity, which makes it excel at breaking the iron dysregulation/neurinflammation cycle [393, 394]. Another interesting action for melatonin is that it can form complexes with ferric iron (Fe^+3^) and hence reduce its reduction to ferrous and the resulting free radicals [395]. In several preclinical studies, melatonin has proven itself worthy in the protection of neurons against iron-induced toxicity [396–399]. Ortega-Gutierrez et al. reported that melatonin enhances the anti-oxidant effect of deferoxamine against lipid peroxidation in rat brain homogenates [400]. Melatonin inhibited NCOA4-mediated ferroptosis in age-related cataracts with higher expression of GPX and H ferritin [401]. In a mouse model of traumatic brain injury (TBI), melatonin exerted an anti-inflammatory and anti-ferroptotic effect through its action on MT2 receptors and upregulation of IL-33 and H ferritin. Interestingly, the protective effect of melatonin was abolished after the deletion of H ferritin, emphasizing the anti-ferroptotic role of melatonin through working on iron homeostasis [402]. Rui et al. reported similar results in a TBI model where melatonin alleviated TBI-induced ferroptosis, associated behavioral deficits, neuronal injury, and cortical volume loss. Melatonin also decreased the number of iron-positive cells, alleviating TBI-induced iron accumulation. However, in H ferritin KO mice, melatonin relinquished its protective effect. On all levels, melatonin exerted a similar outcome to liproxstatin-1, a known ferroptosis inhibitor, making its mark as a genuine ferroptosis inhibitor [74]. In an acute sleep deprivation-associated memory loss model, melatonin decreased memory deficits, iron-positive cells, and dysregulation in iron transporters TfR1, DMT1, and FPN1 [403].

The positive reputation of melatonin as an iron chelator in iron-overload-related disorders is only surpassed by its long-standing reputation as an anti-oxidant. Melatonin tips the scale of the oxidative status in favor of antioxidants, activating multiple antioxidant enzymes, such as GPX4 and SOD, and scavenging for ROS. The direct ROS scavenging capacity of melatonin interferes with the labile iron-induced fenton reaction, minimizing iron-induced damage [404–406] and raising melatonin’s chances as a sophisticated ferroptosis inhibitor. One of melatonin’s key actions as an antioxidant is to promote the function of NrF2 [407]. Through its action on MT2, melatonin activated NrF2 and protected against ferroptosis in a subarachnoid hemorrhage rat model. The blocking of MT2 eliminated melatonin’s protective effect [408]. In radiation-induced neuronal injury and cognitive impairment, melatonin-receiving mice showed improvement on the cognitive level. Melatonin alleviated the underlying ferroptosis in hippocampal neurons by reversing the downregulation of NrF2, GPX4, and SLC7A11 [409]. In a hypoxic ischemic brain damage model, melatonin-receiving rats showed improved memory and learning. Melatonin protected hippocampal neurons against ferroptosis by enhancing the NrF2/GPX4 pathway [410]. Melatonin can act against GSH depletion through its positive effect on the antiporter SLC7A11. As mentioned, melatonin helps the upregulation of SLC7A11 by promoting the function of NrF2 [409, 411–413] and inhibiting the repressor function of p53 on SLC7A11 [414, 415]. By working smarter, not harder, melatonin can decrease the availabilityof PUFA and therefore cut off the fuel for ROS production and lipid peroxidation. Melatonin decreases the expression of ACSL4, which works in favor of decreased incorporation of PUFA in cell membranes [391, 408, 416, 417]. In addition, melatonin protects against ferroptosis by reducing the expression of the H2O2-producing LOX enzymes [414, 418] and has the potential to do so by reducing the expression of the H2O2-producing NOX enzymes [419–422].

The era of SARS-CoV-2 brings the old nuance of the melatonin-angiotensin axis to light. Interestingly, Ag II, which accumulates with COVID-19-mediated degradation of ACE2 receptors [337], works on pinealocytes, activating tryptophan hydroxylase and limiting melatonin synthesis [195]. This goes in line with the remarks about possible deficient melatonin in COVID-19 patients, especially those suffering from insomnia [423]. Also, melatonin happens to interact with ACE2 receptors, changing their interface and capacity to bind to the SARS-CoV-2 spike protein, decreasing brain viral load and inflammation [278]. Ag II-induced ferroptosis was detected in astrocytes [424], vascular endodthelial cells [357], and cardiomyocytes [425]. The antagonistic relationship between melatonin and Ag II was repeatedly displayed, with melatonin playing a protective role against Ag II-induced injury [426–431]. These reports open new prospects for melatonin as a ferroptosis inhibitor through modulating the RAS system and inhibiting Ag II.

Another unique way for melatonin to act as a ferroptosis inhibitor is through its interaction with clock genes. In an ethanol-induced liver injury model, melatonin exerted NrF2-mediated anti-ferroptosis action through the modulation of BMAL1 [432]. Notably, melatonin happens to downregulate the elevated SQSTM1/p62 in ALS and in AD mouse models [254, 433]. These findings promote the hypothesis of melatonin acting against SQSTM1/p62, the cargo receptor that incites autophagic degradation or clockophagy of BMAL1/ARNTL and, by extension, the resulting ferroptosis [374]. Li et al. reported a decreased expression of CLOCK, CRY1, PER1, and PER2 in the peripheral blood mononuclear cells of 326 PD patients compared to their healthy controls, along with a decreased concentration of melatonin [434]. A three-month administration of 25 mg/d of melatonin increased the expression of BMAL1 in the peripheral blood of 26 PD patients in a randomized clinical trial [435]. In aged Wistar rats, the loss of rhythmic expression of BMAL1, Cry1, and Cry2 was reversed by an eleven-day melatonin administration [436]. These studies build up the possibility of melatonin being able to reverse the underling BMAL1 deficiency-induced ferroptosis in post-COVID-19 aging and neurodegeneration.

## Conclusion

Over the years, melatonin’s properties unfolded as a circadian regulator, anti-inflammatory, anti-oxidant, immune modulator, iron chelator, and, lately, an anti-ferroptosis. Melatonin was always in the picture when it came to viral breakouts, and history repeated itself with the gloomy era of COVID-19, with melatonin being, once again, an efficient anti-viral. With the catastrophe of COVID-19 fading away, its aftermath is becoming more evident in the form of accelerated brain aging and neurodegeneration. With its widely reported role in combating the underlying pathologies of aging and multiple neurodegenerative disorders, melatonin becomes such a unique, well-fitting solution for the post-COVID-19 trajectory of aging and neurodegeneration. Another appealing trait of melatonin in such a predicament is its ability to inhibit ferroptosis, which is a strongly proposed underlying mechanism for post-COVID-19 aging and neurodegeneration. COVID-19, aging, and neurodegenerative disorders share five events that end up in ferroptosis, which are neuroinflammation, iron dysregulation, anti-oxidant defense repression, ACE2/Ag II disruption, and clock gene alterations. Luckily, melatonin can tackle all five events, making it the perfect ferroptosis inhibitor against the ferroptosis-induced post-COVID-19 aging and neurodegeneration.

## Data Availability

Not applicable. This manuscript does not contain any data.
